# Immunogenic epitope panel for accurate detection of non-cross-reactive T cell response to SARS-CoV-2

**DOI:** 10.1172/jci.insight.157699

**Published:** 2022-05-09

**Authors:** Aleksei Titov, Regina Shaykhutdinova, Olga V. Shcherbakova, Yana V. Serdyuk, Savely A. Sheetikov, Ksenia V. Zornikova, Alexandra V. Maleeva, Alexandra Khmelevskaya, Dmitry V. Dianov, Naina T. Shakirova, Dmitry B. Malko, Maxim Shkurnikov, Stepan Nersisyan, Alexander Tonevitsky, Ekaterina Khamaganova, Anton V. Ershov, Elena Y. Osipova, Ruslan V. Nikolaev, Dmitry E. Pershin, Viktoria A. Vedmedskia, Michael Maschan, Victoria R. Ginanova, Grigory A. Efimov

**Affiliations:** 1 National Research Center for Hematology, Moscow, Russia.; 2 Faculty of Biology, Lomonosov Moscow State University, Moscow, Russia.; 3 Faculty of Biology and Biotechnology, HSE University, Moscow, Russia.; 4 Shemyakin-Ovchinnikov Institute of Bioorganic Chemistry, Russian Academy of Sciences, Moscow, Russia.; 5 LLC ‘DNKOM’, Moscow, Russia.; 6 I.M. Sechenov First Moscow State Medical University, Ministry of Health of Russia (Sechenov University), Moscow, Russia.; 7 Dmitry Rogachev National Medical Research Center of Pediatric Hematology, Oncology and Immunology, Moscow, Russia.

**Keywords:** COVID-19, Clinical Trials, Antigen, Peptides, T cells

## Abstract

The ongoing COVID-19 pandemic calls for more effective diagnostic tools. T cell response assessment serves as an independent indicator of prior COVID-19 exposure while also contributing to a more comprehensive characterization of SARS-CoV-2 immunity. In this study, we systematically assessed the immunogenicity of 118 epitopes with immune cells collected from multiple cohorts of vaccinated, convalescent, healthy unexposed, and SARS-CoV-2–exposed donors. We identified 75 immunogenic epitopes, 24 of which were immunodominant. We further confirmed HLA restriction for 49 epitopes and described association with more than 1 HLA allele for 14 of these. Exclusion of 2 cross-reactive epitopes that generated a response in prepandemic samples left us with a 73-epitope set that offered excellent diagnostic specificity without losing sensitivity compared with full-length antigens, and this evoked a robust cross-reactive response. We subsequently incorporated this set of epitopes into an in vitro diagnostic Corona-T-test, which achieved a diagnostic accuracy of 95% in a clinical trial. In a cohort of asymptomatic seronegative individuals with a history of prolonged SARS-CoV-2 exposure, we observed a complete absence of T cell response to our epitope panel. In combination with strong reactivity to full-length antigens, this suggests that a cross-reactive response might protect these individuals.

## Graphical abstract

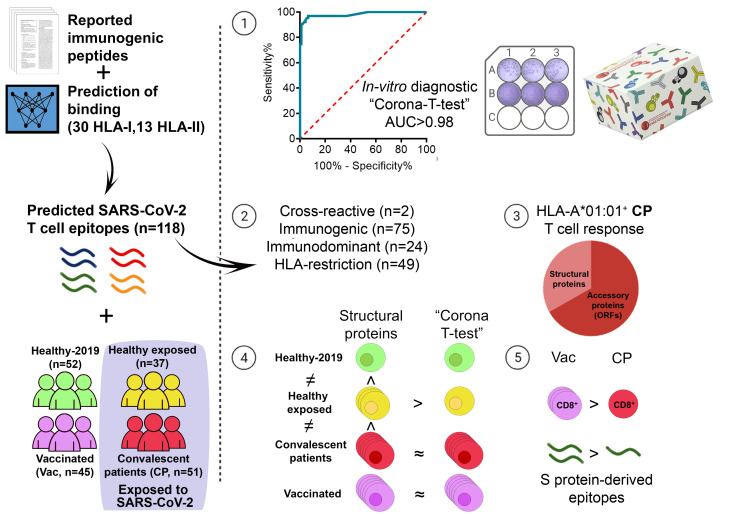

## Introduction

The COVID-19 pandemic has posed a considerable challenge for healthcare systems worldwide, necessitating the rapid development of novel diagnostic tools. Reverse transcription PCR (RT-PCR) is the gold standard assay for confirming COVID-19 infection, while serology tests are commonly used for retrospective diagnosis, assessment of vaccination efficiency, and measurement of the stability of immune protection over time. Nevertheless, estimating the actual rate of infection is complicated because many infections are asymptomatic, and up to 15% of patients do not develop a humoral immune response to infection (1–3)

T cell response can offer an independent metric of SARS-CoV-2–specific immunity in the aftermath of either COVID-19 (4–8) or vaccination (9–13). It has been demonstrated that IgG titers strongly correlate with protection (14–16) and provide it even in the absence of T cells, both in animal models (17, 18) and in prospective human studies (16, 19). However, the studies have suggested that cellular immunity has a role in the context of suboptimal humoral response (16, 18, 19) or at the early stages after vaccination before seroconversion (20, 21). It has also become clear that the humoral response gradually fades and may no longer be detectable 6 months after infection (22, 23) or after vaccination (24), whereas T cells persist long after exposure (23, 25, 26). Indeed, T cell responses have remained detectable for up to 17 years after infection with SARS-CoV-1 (27).

However, the detection of SARS-CoV-2–specific T cell response is hindered by the relatively frequent occurrence of false-positive responses in individuals not exposed to SARS-CoV-2 due to cross-reactivity to other coronaviruses (4, 7, 28). The role of this response remains controversial; some studies report that such low-affinity cross-reactive responses may contribute to a poor prognosis (29), while others have demonstrated that preexisting memory T cells rapidly respond upon vaccination (30) and that the expansion of cross-reactive T cells is associated with mild disease (31) and may explain asymptomatic infections (32). Nevertheless, it is important to distinguish between cross-reactive and COVID-19–specific T cell responses. To date, several kits for in vitro detection of T cell response have been proposed based on ELISpot/FluoroSpot technology (33–36), high-throughput sequencing–based (HTS-based) detection of T cell receptor (TCR) sequences (37), and measurement of cytokine production in whole blood (38). Most of these exploit custom peptide sets that were bioinformatically selected to minimize cross-reactivity, but to the best of our knowledge, these were not experimentally validated on prepandemic samples.

Previous studies have predicted (39) and experimentally confirmed (37, 40–42) numerous SARS-CoV-2 T cell epitopes. Some researchers have focused on the properties of individual epitopes, such as the diversity and repertoires of specific TCRs and structural aspects of epitope recognition (7, 43–48). Others have aimed, rather, at characterizing the response to sets of epitopes (26, 48–50). Nevertheless, the employment of different assays hinders direct comparison, and the limited cohort size and number of epitopes tested per study have left essential questions pertaining to the immunodominance of individual epitopes — and, for some epitopes, their HLA restriction — unresolved.

Understanding patterns of immunodominance of SARS-CoV-2 epitopes could guide future vaccine development. For example, ORF1ab- and ORF3a-derived epitopes seem to be more immunogenic than other components of the viral proteome — including the S glycoprotein — in individuals bearing HLA-A*01:01, which is common in the European population (37, 40, 49). Moreover, although several studies have demonstrated that the total magnitude of CD8^+^ and CD4^+^ T cell response in people vaccinated with existing S protein–based vaccines is on par with or even surpasses that of patients who have recovered from infection (9–11, 13), it remains unclear whether the spectrum of recognized epitopes is the same in both groups. Given that increased diversity of recognized epitopes is known to correlate with better outcomes in some other viral infections, such as with hepatitis B virus (51), it is important to profile T cell immunity — including the landscape of recognized epitopes — in vaccinated individuals and patients after natural infection with SARS-CoV-2.

In the present study, we aimed to systematically characterize a preselected set of 118 SARS-CoV-2 epitopes presented by common HLA-I and -II alleles. In sharp contrast to full-length antigens, the selected epitopes did not induce a response in prepandemic samples, with the exception of 2 HLA-II–restricted peptides. We confirmed immunogenicity for 75 epitopes and HLA restriction for 49 of them, including 9 HLA-I epitopes that had previously displayed ambiguous binding. We further demonstrated that 7 HLA-I and 7 HLA-II epitopes are presented by more than 1 HLA allele. Twenty-four epitopes were immunodominant, meaning they were identified in at least 50% of patients with the restricting HLA allele. Based on these findings, we designed the ELISpot-based in vitro diagnostic Corona-T-test for specific detection of COVID-19– or vaccine–induced — but not cross-reactive — T cell response to SARS-CoV-2. This test demonstrated 95% accuracy in a clinical trial of 119 immunized (vaccinated or convalescent) and 101 unexposed individuals. We subsequently used this test to study a cohort of asymptomatic seronegative individuals with a history of prolonged SARS-CoV-2 exposure and observed a lack of specific T cell response combined with strong response to full-length antigens. Finally, we demonstrated that individuals vaccinated with the 2-component Gam-COVID-Vac adenoviral vaccine (Sputnik-V) recognized a significantly higher number of S-derived CD8^+^ epitopes in comparison with the convalescent cohort.

## Results

### Identifying a set of potential immunogenic and non-cross-reactive T cell epitopes.

**Figure 1:**
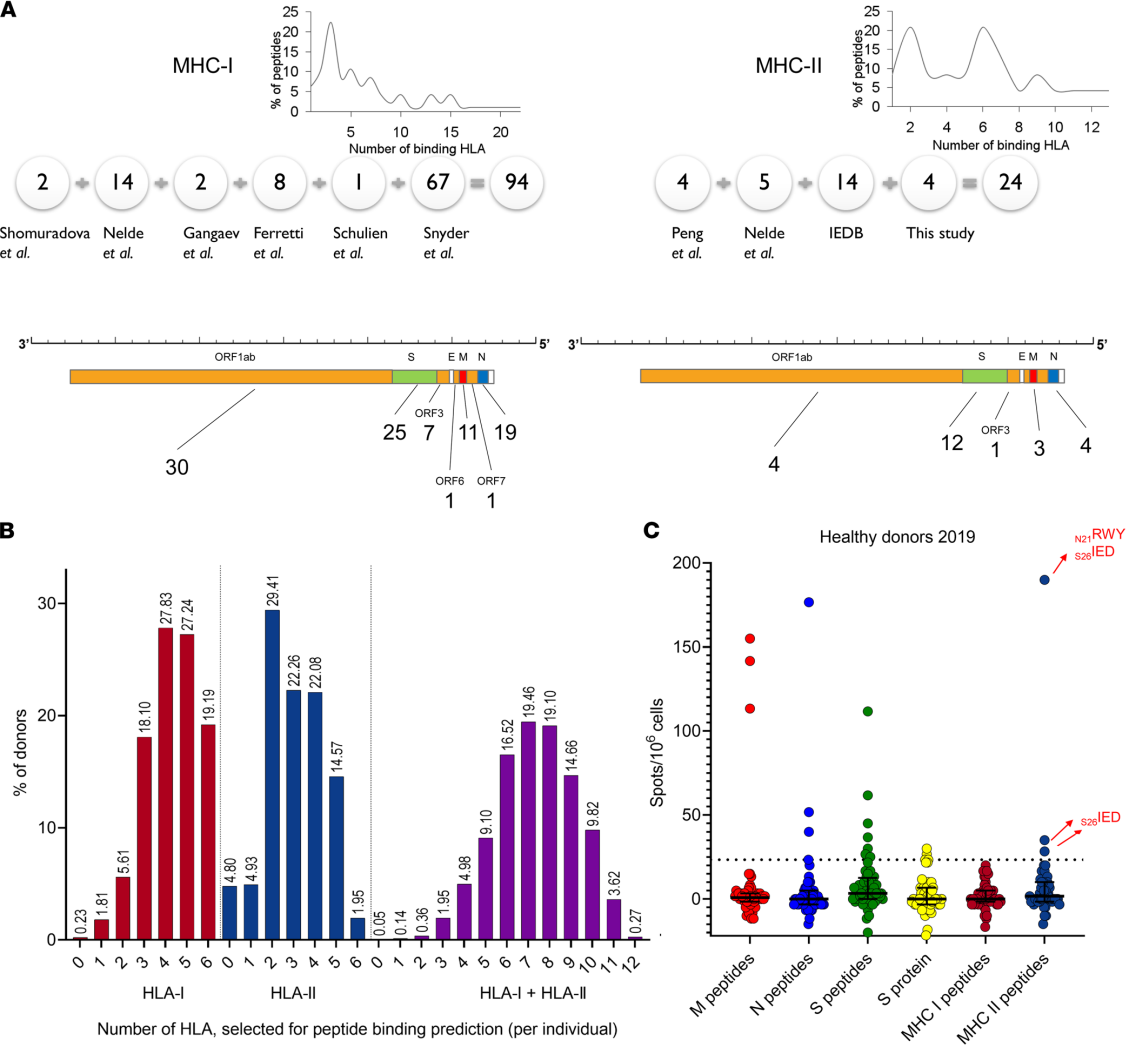
Characteristics of the peptide set. (**A**) Number of epitopes selected from each indicated publication (detailed in Supplemental Table 1) for the MHC-I (left) and -II (right) sets. The distribution of the peptides according to the number of HLA that they bind is shown at top. The *x* axis displays the number of predicted binding alleles per peptide. The *y* axis shows the percentage of peptides that bind to a given number of alleles. Numbers below the SARS-CoV-2 genome schematic indicate the number of peptides derived from each gene. (**B**) The number of HLA class I (left) and -II (middle) alleles alone or in combination (right) that are predicted to bind at least 1 peptide from the set per individual among 2210 donors from the BM registry. (**C**) Antigen response among the healthy (HD-2019) cohort (*n* = 52). The normalized mean of 2 duplicate wells and the median and interquartile range. Cross-reactive MHC-II peptides are marked with red arrows. The positive threshold is indicated by the dotted line.

To assemble a set of peptides, we collected available information on SARS-CoV-2 T cell epitopes (7, 37, 40–42, 48, 50), as depicted in Figure 1A. The selected peptides were derived from both structural and nonstructural SARS-CoV-2 proteins, based on their high immunogenicity in COVID-19 convalescent patients and their low immunogenicity in nonexposed individuals, as well as their predicted binding to one or several common HLA alleles across the European population. The final set included 94 predicted HLA-I binders (i.e., MHC-I peptides) and 24 predicted HLA-II binders (i.e., MHC-II peptides), where each MHC-I and MHC-II peptide was predicted to bind, on average, to 4 HLA-I and 5 HLA-II alleles, respectively (Figure 1A and Supplemental Table 1; supplemental material available online with this article; https://doi.org/10.1172/jci.insight.157699DS1). We did not observe a difference in the homology scores of the selected peptides and a set of other SARS-CoV-2 immunogenic epitopes annotated in the Immune Epitope Database (IEDB) relative to common cold coronaviruses (Supplemental Figure 1, A–D). To estimate the theoretical coverage of the population for this set of peptides, we evaluated the frequency distribution of the restricting HLA alleles among HLA-typed individuals in the local BM donor registry (*n* = 2210). Only a single person (0.05%) had none of the alleles that were predicted to present these MHC-I or MHC-II peptides (Figure 1B), indicating the designed peptide set offers sufficient predictive sensitivity.

### Full-length antigens induce a cross-reactive response compared with the selected peptides.

We compared the specificity for pools of peptides spanning the full length of the various SARS-CoV-2 structural proteins — S protein (S), nucleoprotein (N), and membrane protein (M) — with that for the MHC-I and -II peptide sets using a cohort of prepandemic healthy donor samples (HD-2019; *n* = 52) and measuring the IFN-γ response by ELISpot. Ten donors produced a positive (≥23.31 spots/1 × 10^6^ PBMC) T cell response to any of the peptide pools (S, N, or M) (Figure 1C) or to recombinant S protein. Three of these donors also had a positive response to the set of MHC-II peptides (Supplemental Figure 1E). Using matrix pools, we identified 2 cross-reactive MHC-II peptides (_N21_RWY from N protein and _S26_IED from S protein; Figure 1C and Supplemental Figure 1F; sequences are given in Supplemental Table 1). The high frequency of cross-reactive responses induced by the S, N, and M peptide pools or by recombinant S protein makes these targets ill suited for measuring SARS-CoV-2–specific T cell response.

### Diverse response in convalescents versus MHC-I–focused response in vaccinated individuals.

**Figure 2:**
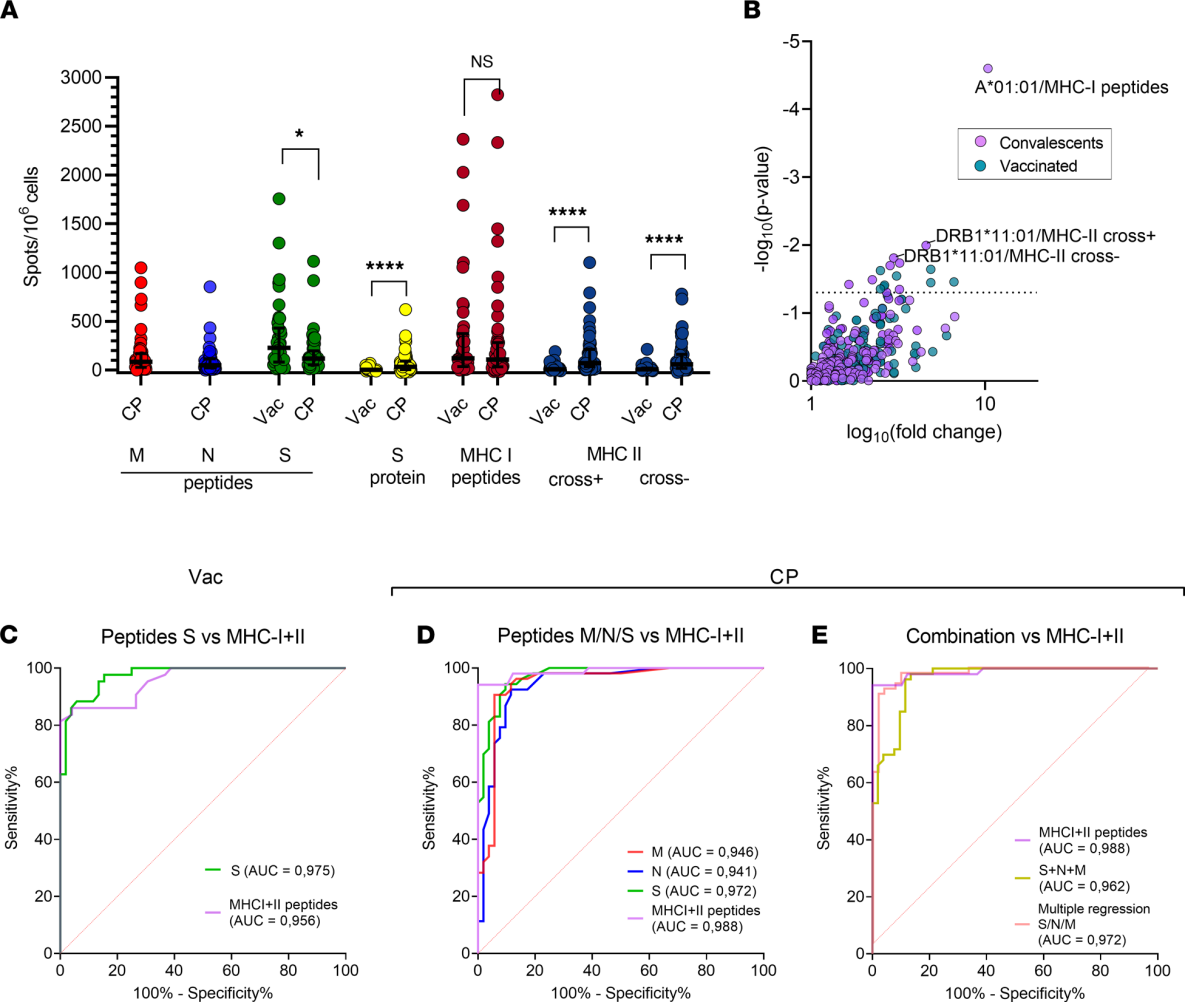
Response to MHC-II peptides differs significantly in Vac and CP donors. (**A**) Response to the indicated antigens as measured by ELISpot for Vac (*n* = 43) and CP (*n* = 51). A normalized mean of 2 duplicate wells and a median with interquartile range. Mann-Whitney *U* test (S peptides, *P* = 0.013; MHC-II peptides, recombinant S protein, *P* < 0.0001). (**B**) Volcano plot shows the effect of a particular HLA allele on response to the same peptide sets and antigens. The *x* axis denotes the decimal logarithm of the ratio of the median response among HLA carriers to that of individuals without the HLA. The *y* axis denotes the negative decimal logarithm of the *P* value. The 3 most significant associations are annotated. *P* = 0.05 is depicted by the dotted line (Fisher’s exact test). (**C**–**E**) Receiver operating characteristic (ROC) curves for MHC I + II peptides versus S peptides in Vac versus HD-2019 samples (**C**); MHC I + II peptides versus S, N, or M peptides in CP versus HD-2019 samples (**D**); and MHC I + II peptides versus the sum of S, N, and M peptides and versus multiple regression model, incorporating S, N, and M peptides in CP versus HD-2019 samples (**E**). Three HD-2019 donors with cross-reactive responses were excluded from the ROC analysis for MHC I + II peptides.

We next analyzed the response to S, N, and M peptides and MHC-I and MHC-II peptides in cohorts of convalescent patients (CP; *n* = 51) and Sputnik-V–vaccinated individuals (*n* = 45, Vac) using ELISpot. Full information on these cohorts is provided in Supplemental Table 2. We excluded the 2 cross-reactive peptides identified above (_N21_RWY and _S26_IED) to create a new MHC-II cross^–^ peptides set; the initial MHC-II set will subsequently be referred to as MHC-II cross^+^. In agreement with recently published research (13, 35), we observed that Vac individuals demonstrated a greater response to S peptides than CP, while the response to N and M peptides in Vac was nonexistent (Supplemental Figure 3A). Both cohorts demonstrated comparable responses to the MHC-I set (Figure 2A), although peptides derived from S protein accounted for only 27% of that set.

Surprisingly, individuals in the Vac group demonstrated a significantly weaker response to MHC-II peptides in comparison with CP (Figure 2A). We hypothesized that the MHC-II peptide set, although it included multiple S-derived peptides, was skewed toward immunogenic peptides from the other antigens, N and M. We tested the reactivity to MHC-II peptides derived from S protein (excluding _S26_IED peptide) in 8 CP donors with the strongest response to the MHC-II peptides (>166.5 spots/1 × 10^6^ PBMC). We observed a detectable response to S-derived epitopes in a single donor (Supplemental Figure 2A). This suggests that S-derived MHC-II epitopes might be nonimmunogenic or evoke a low-frequency T cell response that is barely detectable by ELISpot without ex vivo expansion. We also observed negligible response in the Vac cohort to the recombinant S protein (Figure 2A) in comparison with CP, where this response was more strongly correlated with the response to MHC-II peptides than to MHC-I peptides (Supplemental Figure 2B). This probably reflects the predominant presentation of the recombinant protein by the MHC-II pathway. We tested whether time influenced the antigen response within our sampling period, and we did not observe a significant association with the recombinant S protein, MHC-I, or MHC-II peptides (Supplemental Figure 2, C–H).

Based on the sum of positive spots in wells with MHC-I and MHC-II cross^–^ peptides (MHC I + II) we made the observation that the response to the limited number of MHC I + II peptides was of a similar magnitude as the sum of responses to S, N, and M peptides in CP cohorts (Supplemental Figure 3A). This effect was replicated in the Vac cohort, where the response to the MHC I + II set was not different from the response to the S peptides. Notably, several Vac donors demonstrated even greater responses to the MHC I + II set in comparison with S peptides. We next assessed the contribution of cross-reactive peptides _S26_IED and _N21_RWY to the total response (Supplemental Figure 3B). We observed a significant impact of these peptides (>99.9 spots/1 × 10^6^ PBMC) in 3 CP donors, but this response was only present in patients with high reactivity to other MHC-II peptides. Patients with low responses to these cross-reactive peptides demonstrated virtually no difference between MHC-II cross^+^ and cross^–^ sets, and therefore lower sensitivity due to the exclusion of the _S26_IED and _N21_RWY peptides is unlikely.

We next examined the effect of a particular HLA allele on the magnitude of response to different antigens (Figure 2B). For CP donors, we observed a very strong association between the presence of HLA-A*01:01 and the number of spots in response to MHC-I peptides. Indeed, most of the HLA-A*01:01–restricted peptides, which we further confirmed as immunodominant, were derived from viral ORFs and, thus, could not evoke a response in the Vac cohort. This suggests that HLA-A*01:01 is associated with increased response to ORF-derived epitopes (Supplemental Figure 3C). The increased response to MHC-II peptides that we observed in carriers of DRB1*11:01 may be associated with higher immunogenicity of some peptides in the context of this particular allele. For the other HLA alleles, we observed significant variability in the responses. We also compared differences in the prevalence of common HLA alleles (>10% phenotypic prevalence) between groups and observed that the CP cohort included fewer patients with B*07:02/C*07:02 (Supplemental Figure 3D) in comparison with either BM donors or the Vac cohort.

### The selected set of peptides demonstrated high diagnostic accuracy.

We then assessed the diagnostic accuracy of the selected peptide set. For Vac donors, surprisingly, MHC I + II peptides demonstrated a very similar AUC compared with S peptides (0.96 versus 0.98), suggesting that the limited number of S protein–derived peptides is sufficient for detecting T cell response here (Figure 2C). In contrast, the MHC I + II peptides discriminated CP from HD-2019 better than any of the peptide pools covering full-length antigens (Figure 2D), providing the same sensitivity (94%) with better specificity (S [88%, cutoff 26.64 spots/1 × 10^6^ PBMC] versus MHC I + II [94%, cutoff 25 spots/ 1 × 10^6^ PBMC]) and a larger AUC (0.99 versus 0.97). In the CP cohort, the lack of specificity was more prominent when we analyzed the sum of spots in wells with M, N, and S peptides (Figure 2E); this reduced specificity could not be fully mitigated by application of the logistic regression model based on the spot values for the S, N, and M peptides. However, the wide confidence intervals of AUC values do not allow us to assess statistical significance for these differences.

### Profiling HLA presentation of MHC-I and -II epitopes.

**Figure 3:**
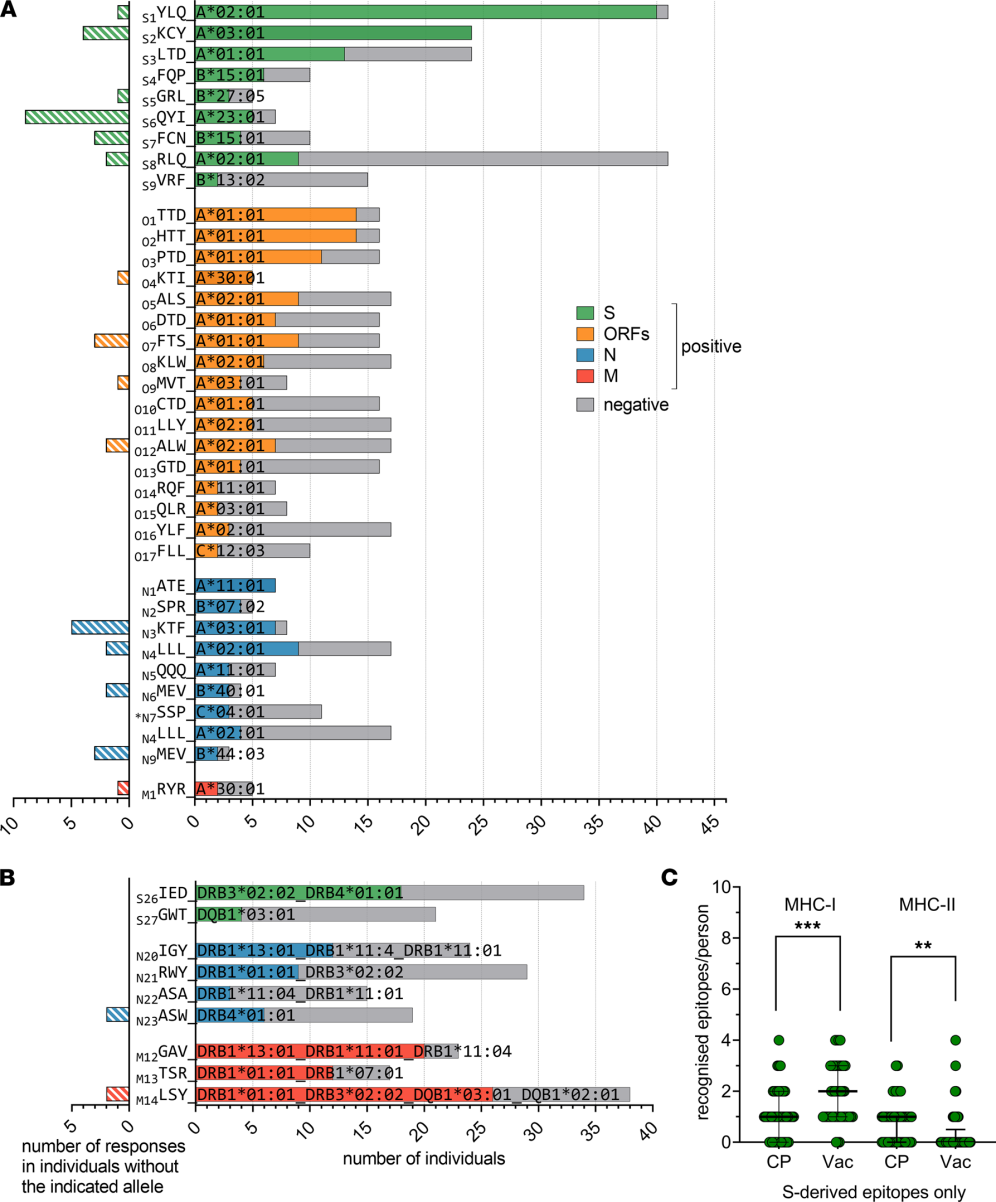
T cell response to MHC-I and MHC-II epitopes in CP and Vac. (**A** and **B**) Immunogenicity in carriers of various HLA-I (**A**) or HLA-II (**B**) alleles. For the right sides of the plots, gray bars indicate the number of tested carriers; colored bars indicate the number of responses. Colors indicate source protein, with 3-letter amino acid codes at left. Superscript numbers indicate peptide length, and the asterisk denotes a peptide with an ambiguous HLA association. The left sides of the plots show the number of responses in donors without the indicated HLA. For HLA-I, the association between the response and a single HLA allele is shown; for HLA-II, the best associations (including associations with several HLAs) are shown. Fisher’s exact test; *P* < 0.05 were considered significant (exact *P* values are specified in Supplemental Table 3). (**C**) Number of S protein–derived MHC-I and MHC-II epitopes recognized per individual for the CP and Vac cohorts; median and interquartile range are shown

In order to systematically analyze the immunogenicity and HLA restriction of each epitope in our set, we performed a short-term memory T cell expansion assay. Peripheral blood mononuclear cells (PBMCs) were stimulated with the complete set of MHC-I and -II peptides (split into 5 expansion pools), followed by ~10 days of culturing. To reduce the number of individual peptides tested in each assay, we assessed the response to smaller pools of about 5 peptides each using IFN-γ ELISA. When a response to one of these ELISA pools was detected, the expansion was tested for reactivity to the individual peptides in that pool, although only peptides that bound to that individual’s HLA alleles were tested. Supplemental Table 2 summarizes all of the epitopes that were tested individually or as part of ELISA pools. We observed at least 1 response to 59 of 94 MHC-I epitopes and at least 2 responses to 47 epitopes. The latter epitopes were selected for HLA association analysis. Using the Fisher’s exact test, we identified the association with a particular HLA for 36 of the 47 immunogenic MHC-I epitopes (Figure 3A). Of those, 22 epitopes were exclusively immunogenic in carriers of the associated HLA allele, with no off-HLA response. For 4 peptides, 1 off-HLA response was detected, and 10 peptides demonstrated more than 1 off-HLA response. For this latter set of peptides, we searched for a second allele association that covered most of the off-HLA responses (Supplemental Figure 4A). For the remaining 11 peptides that did not demonstrate association with any particular HLA allele, we sought associations with a combination of 2 HLA alleles. We identified such a combination for 2 peptides (_S10_ADA and _N10_KAY; Supplemental Figure 4A), and we observed no significant association for the remaining 9 peptides, suggesting that they may bind 3 or more alleles (e.g., _M2_NRF presumably binds B*08:01, C*04:01, and C*06:02; Supplemental Table 2) or are insufficiently immunogenic. The _N7_SSP peptide could not be definitely assigned, as it demonstrated the association with either C*04:01 (Figure 3A) or B*35:02 and B*35:01 (Supplemental Figure 4A). We also detected 2 MHC-I epitopes within predicted MHC-II epitopes.

We observed at least 1 response for 16 of 24 MHC-II epitopes. For the 13 epitopes demonstrating at least 2 CD4^+^ responses, we could not detect any apparent association with a single HLA-II allele (Supplemental Figure 4B). After searching for associations with a combination of 2 HLA alleles, several peptides still demonstrated multiple off-HLA responses (Supplemental Figure 4B), suggesting an association with 3 or even 4 HLA alleles. For these peptides, we present the final HLA association assignment that explained most of the responses (Figure 3B). Statistics and association patterns are presented in Supplemental Table 3. HLA alleles with a validated association with at least 1 immunogenic peptide are further referred to as confirmed HLA.

Moreover, 19 MHC-I and 5 MHC-II epitopes were immunodominant, in that they were recognized in at least 50% of patients with the restricting HLA alleles. The most dominant MHC-I epitopes from S protein were _S1_YLQ/A*02:01 and _S2_KCY/A*03:01, with nearly 100% response; in contrast, _S2_KCY produced a response in only 4 of 12 A*11:01 carriers. We also noted that _S6_QYI generated higher immunogenicity with A*23:01, in comparison with the already observed association with A*24:02 (45). Among ORF-derived epitopes, _O1_TTD/A*01:01, _O2_HTT/A*01:01, and _O4_KTI/A*30:01 elicited the most frequent response. _N1_ATE/A*11:01, _N2_SPR/B*07:02, and _N3_KTF/A*03:01/A*30:01 from N protein also showed a dominant response. In contrast to _S2_KCY, _N3_KTF was equally immunogenic, both in A*03:01 and A*30:01. Assessment of immunodominance for MHC-II epitopes was hindered by their promiscuous binding, but _S26_IED, _M12_GAV, _M13_TSR, _M14_LSY, and _N20_IGY may be considered immunodominant in the context of all of the HLAs they bound. By analyzing 12 SARS-CoV-2 variants of concern (VOC), including the Omicron variant, we observed that 9 immunogenic epitopes are affected with mutations (Supplemental Table 1). Importantly, the most frequently mutated epitopes were only minimally immunogenic (Supplemental Table 1 and Figure 3, A and B), suggesting that SARS-CoV-2 evolution drives evasion, rather, from humoral than from T cell response. In the Omicron, variant only 2 immunogenic epitopes were mutated. Only 1 immunodominant epitope (_S4_FQP/B*15:01) was recurrently mutated.

### Broader repertoire of recognized S protein–derived MHC-I epitopes in vaccinated individuals compared with convalescents.

Unsurprisingly, expanded T cells from the CP cohort recognized a higher number of MHC-I epitopes than those from Vac donors (median 4.5 versus 2), in which the response was limited to the S protein (Supplemental Figure 4C). However, this decrease in the number of recognized epitopes was not accompanied by a decrease in overall response magnitude (Figure 2A), suggesting a more robust response per epitope. At the same time, the number of recognized MHC-I epitopes from S protein was significantly higher in the Vac cohort than in CP (median 2 versus 1) (Figure 3C). In contrast, the Vac cohort exhibited significantly fewer recognized MHC-II epitopes per person, both for all MHC-II epitopes (median 3 versus 0; Supplemental Figure 4C) or S-derived epitopes (median 1 versus 0; Figure 3C). Moreover, using flow cytometry (gating strategy in Supplemental Figure 4E), we confirmed that, in the Vac cohort, most of the responses of expanded T cells to S-derived MHC-II epitopes were mediated by the CD8^+^ rather than CD4^+^ compartment (Supplemental Figure 4D). Accordingly, the profile of recognized MHC-II epitopes was different in Vac and characterized by the common recognition of _S29_LQT and _S30_QQL peptides by CD8^+^ cells (Supplemental Figure 5). We observed a strong prevalence of either B*40:01 or B*44:03 among the CD8^+^ responders to _S30_QQL (4 of 5). A 9-mer peptide AEIRASANL predicted to be a strong binder for both these alleles may explain CD8^+^ reactivity to this peptide. Among 8 responders to the _S29_LQT epitope, 7 were carriers of either A*23:01 or A*24:02. Enclosed 9-mer TYVTQQLI is predicted as a strong binder for both alleles. Another MHC-I epitope — _S9_VRF/B*13:02 — induced response in 2 Vac, but in none of the CP, despite the similar number of B*13:02 carriers in both groups. These data suggest that the observed higher number of recognized MHC-I S protein–derived epitopes per person in Vac compared with CP is either a consequence of a wider repertoire of recognized MHC-I S protein–derived epitopes or superior detection with our readout due to a higher frequency of specific T cells.

In contrast, although T cells specific to S protein–derived MHC-II peptides could not be detected with ELISpot among PBMCs from either Vac or CP individuals (Figure 2A and Supplemental Figure 2A), they were readily detected after ex vivo expansion in CP but not in Vac samples (Figure 3C and Supplemental Figure 4D). We did not observe any difference in the prevalence of frequent HLA-II alleles between CP and Vac groups (Supplemental Figure 3D), explaining the striking difference in CD4^+^ T cell response. We hypothesize that the frequency of IFN-γ–producing S protein–specific CD4^+^ T cells in the Vac cohort has fallen below the detection threshold by the median of 66 days after primary vaccination (45 days after booster). And in contrast to MHC-I epitopes, the absence of the other immunogens besides S protein did not widen the CD4^+^ response to the S protein, at least within the given timeframe of sampling. The CP cohort was sampled at median 41 days after symptoms (Supplemental Table 2), which may explain higher frequency of the IFN-γ–producing CD4^+^ T cells in comparison with Vac.

We also observed responses to non–S-derived epitopes in 10 Vac donors. In 2, we observed response exclusively to cross-reactive _N21_RWY or _N2_SPR epitopes, while 8 responded to 2 or more non–S protein epitopes, suggesting prior infection.

### Clinical confirmation of the diagnostic accuracy of the final peptide set.

**Figure 4:**
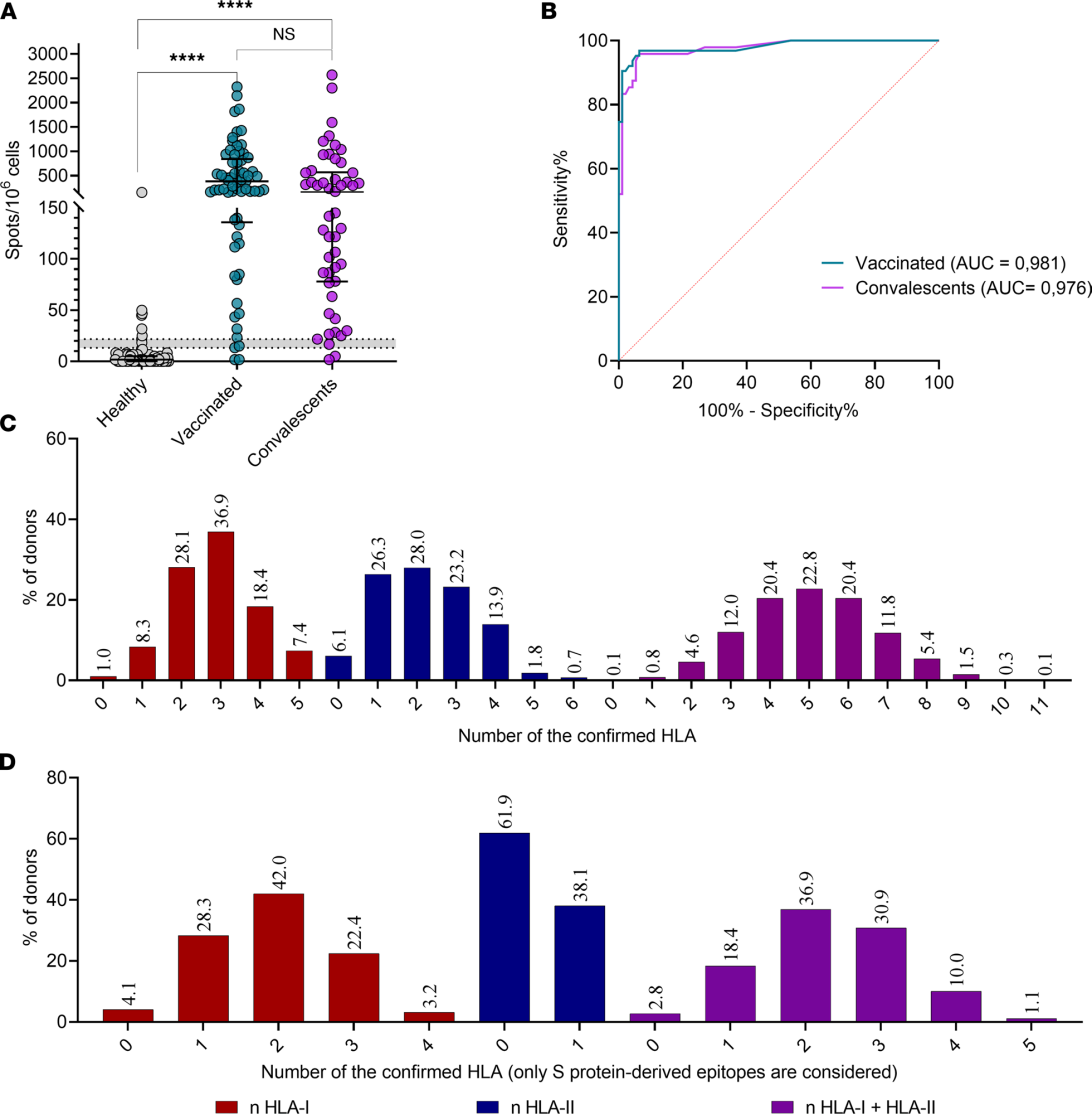
Clinical trial confirmed the high accuracy of the Corona-T-test in discriminating healthy donors from vaccinated and convalescents. (**A**) Scatter plot shows Corona-T-test result. A normalized mean of 2 duplicate wells for the Vac_trial (*n* = 69) and CP_trial (*n* = 50) and HD-2021 (*n* = 95) cohort with median and interquartile range. Results in the gray zone (*n* = 6) were excluded from the ROC-analysis. Kruskal-Wallis with Dunn’s post hoc test; ****P < 0.0001. (**B**) ROC curve for Vac_trial (AUC = 0.98) an CP_trial (AUC = 0.97) versus HD-2021_trial. (**C** and **D**) Number of confirmed HLA-I and -II alleles per individual binding at least 1 peptide from any protein (**C**) or S protein (**D**) (*n* = 2210).

We designed and manufactured an ELISpot-based in vitro diagnostic Corona-T-test for detection of SARS-CoV-2–specific T cells, which included the above-identified combination of peptides (designated here as MHC I + II_IVD). We performed a clinical trial (NCT05165719) in which we enrolled 3 independent cohorts of vaccinated (Vac_trial, *n* = 69), convalescent (CP_trial, *n* = 50), and healthy but unvaccinated individuals (HD-2021, *n* = 101). We measured the T cell response in parallel with the Corona-T-test and by stimulation with MHCI + II_IVD peptides, followed by intracellular IFN-γ staining. As expected, intracellular INF-γ produced a low signal/noise ratio, and multiple patients with a robust ELISpot response were not positive based on intracellular IFN-γ staining (Supplemental Figure 6A). Using flow cytometry, we confirmed that the CD8^+^ response to MHC I + II_IVD was higher than the CD4^+^ response in the Vac_trial and CP_trial cohorts (Supplemental Figure 6B), which is consistent with our previous ELISpot data (Supplemental Figure 3A). We did not observe a correlation between sampling time and humoral or T cell response in the Vac_trial cohort (Supplemental Figure 6, C–E), which is probably due to the short timeframe that had elapsed since the boost vaccination (7–21 days). For CP individuals, we observed a weak association between sampling time and T cell or humoral response (Supplemental Figure 6, E and F). Consistent with the previous results, our Corona-T-test demonstrated high overall accuracy in discriminating HD-2021 from Vac_trial (AUC = 0.98) and CP_trial (AUC = 0.98) (Figure 4, A and B) individuals, with 96.4% sensitivity, 93.5% specificity, and 95% accuracy. Several false positives in the HD-2021 cohort might be explained either by the enrolment of asymptomatic convalescents without detectable antibody response or by the presence of cross-reactive epitopes in our MHCI + II_IVD peptide set. HLA genotyping of the responders in the HD-2021 cohort did not reveal any obvious HLA bias (e.g., high B*07:02 prevalence), most likely excluding the possibility that a single cross-reactive epitope caused the occasional false-positive responses.

We also HLA genotyped the nonresponders (below the gray zone in Figure 4A) from the Vac_trial and CP_trial cohorts to assess possible biases and identify HLA alleles associated with weak response. Among 2 nonresponders in Vac_trial, we observed only 1–2 confirmed HLA alleles presenting epitopes with modest immunogenicity. Two CP_trial nonresponders bore 3 confirmed HLA alleles, which potentially bound 13 and 6 confirmed peptides, respectively. This is comparable with the median number of recognized epitopes per person in the CP group (Supplemental Figure 4C). Thus, we expect that lower responses, at least in CP individuals, are an intrinsic property rather than a consequence of lacking confirmed HLA alleles. In line with this hypothesis, we observed only a modest correlation between the number of confirmed HLA-I alleles and the response to MHC-I peptides in the Vac cohort (*r* = 0.52, *P* = 0.0004) and the number of confirmed HLA-II alleles with MHC-II cross^–^ in the CP (*r* = 0.4, *P* = 0.007) cohort. We reexamined the healthy population (same as in Figure 1B) according to the number of confirmed HLA alleles. Up to 2.8% of the population lacked HLA with confirmed binding of S-derived epitopes, whereas 0.1% lacked any of the HLA alleles confirmed to present SARS-CoV-2 peptides (Figure 4, C and D).

### Healthy SARS-CoV-2–exposed individuals demonstrated strong response to structural proteins but no specific response to SARS-CoV-2.

**Figure 5:**
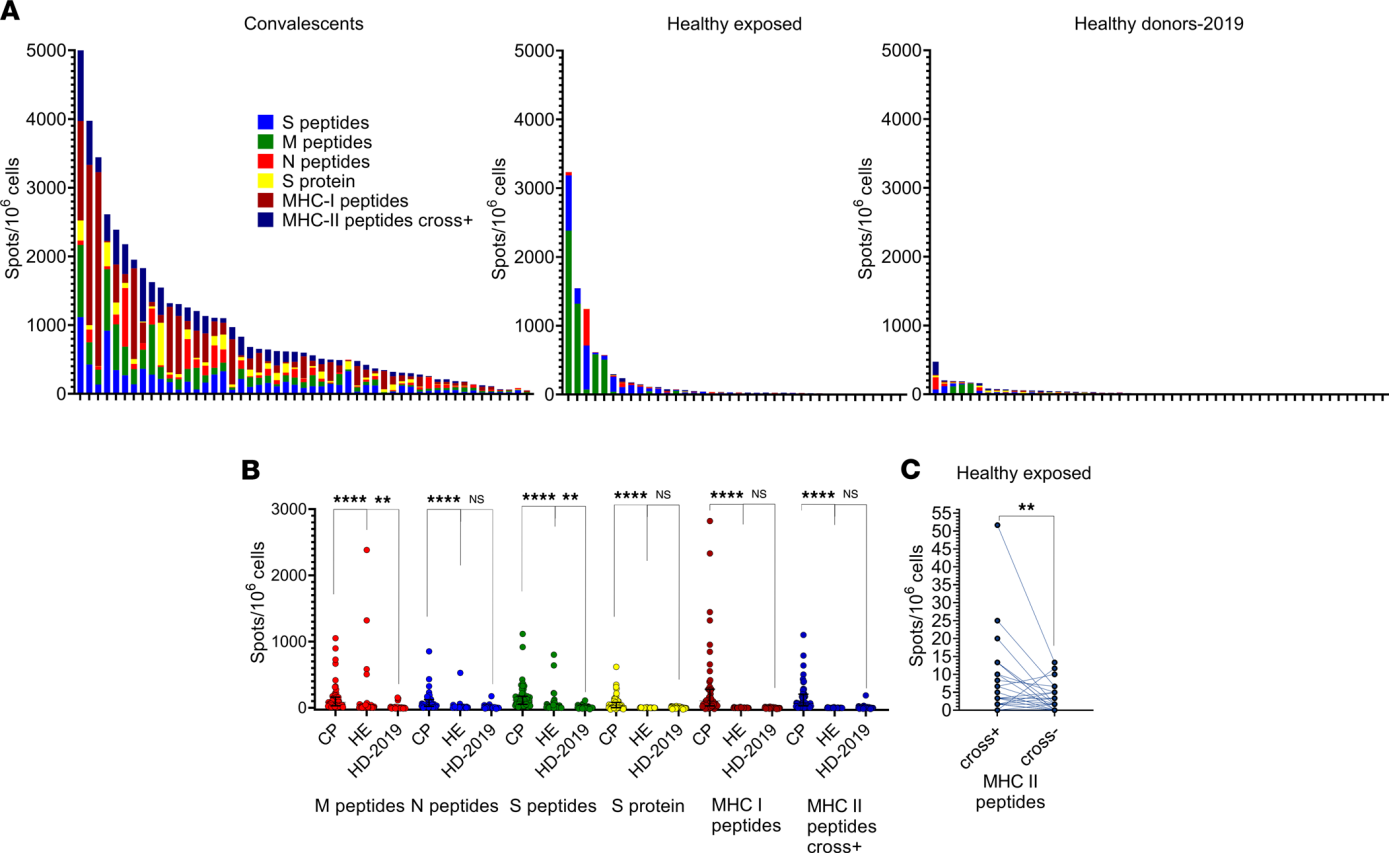
Evidence for a cross-reactive rather than SARS-CoV-2–specific response in the healthy exposed (HE) cohort. (**A**) Response to the antigens (normalized mean of 2 wells after subtracting negative control) in HE (*n* = 37), CP (*n* = 51), and HD-2019 (*n* = 52) cohorts. Each bar represents an individual donor; colors represent particular antigens. (**B**) Comparison of the response to antigens between cohorts as measured with ELISpot. A normalized mean of 2 duplicate well, and the median response and interquartile range. Kruskal-Wallis with Dunn’s post hoc test; ****P < 0.0001, **P < 0.01. (**C**) Difference in responses to MHC-II peptides before and after exclusion of 2 cross-reactive peptides in the HE cohort (Wilcoxon test; *P* = 0.0045).**P < 0.01.

During June–July 2020, we recruited a cohort of healthy exposed (HE) individuals (*n* = 37) who were in close contact with COVID-19 patients but did not report any symptoms and remained IgG^–^ and IgM^–^. The median time of contact was 22 days (IQR, 10–29 days). We did not observe a significant difference between HLA distribution in the HE cohort and CP or BM donors, except for the already observed low frequency of B*07:02/C*07:02 among CP (Supplemental Figure 7). In comparison with HD-2019, HE individuals demonstrated significantly higher responses to M and S peptides. However, no response to MHC-I and MHC-II cross^–^ peptides was detected (Figure 5, A and B). For CP individuals, in contrast, we observed high concordance of positive responses (≥23.33 spots/1 × 10^6^ PBMC) to M, N, or S peptides and MHC I + II peptides (Supplemental Figure 2B and Supplemental Figure 3A), with only 2 of 51 demonstrating a discordant response. Finally, we observed a significant cross-reactive response to the _N21_RWY and _S26_IED epitopes in the HE cohort that was not seen in the Vac cohort (Figure 5C and Supplemental Figure 3B). These results show that the HE cohort is substantially different from both CP and HD-2019, suggesting the presence of a cross-reactive T cell response that might have prevented the development of symptomatic disease and T cell priming with other SARS-CoV-2 epitopes from our set.

## Discussion

Systematic study of the immunogenicity of SARS-CoV-2 T cell epitopes on a large cohort opens the door to vaccine optimization and the utilization of T cell response as an independent measure of immune protection. Here, we have selected a set of immunogenic and putatively non-cross-reactive SARS-CoV-2 peptides that are predicted to bind common HLA-I and -II alleles. Based on analysis of IFN-γ response in 52 prepandemic samples (HD-2019), we identified 2 cross-reactive MHC-II epitopes. MHC-I epitopes (_N2_SPR, _O8_KLW, and _O11_LLY) that have subsequently been proven to be cross-reactive by others (32, 41, 44, 52) did not produce a measurable response in our assay. This could possibly be explained by the low peripheral frequency of the epitope-specific T cells. In support of that hypothesis, HLAs presenting these cross-reactive epitopes were not enriched in a small subgroup of healthy donors (HD-2021) who were positive based on our Corona-T-test.

We compared the MHC I + II peptides to commonly used peptide pools spanning the full-length S, N, and M proteins and to the recombinant S protein. The response to the limited number of peptides in the MHC I + II set was of a similar magnitude as the sum of responses to S, N, and M peptides in both Vac and CP cohorts. Surprisingly some vaccinated individuals demonstrated an even higher response to MHC I + II peptides than to S peptides, underlining the superior immunogenicity of precise epitopes compared with 15-mer peptides, which require trimming before HLA binding. Moreover, all full length, antigens sporadically induced robust responses in prepandemic samples, which makes them ill suited for measuring SARS-CoV-2–specific T cell responses.

We used the MHC I + II peptides identified here as the basis for the ELISpot-based Corona-T-test. This test exhibited 95% accuracy in detecting SARS-CoV-2–specific T cell response in a blinded clinical trial. Intracellular IFN-γ staining performed in parallel was less sensitive but allowed us to confirm our previous finding that the measured IFN-γ response was largely mediated by CD8^+^ cells. HLA genotyping of nonresponders from the Vac_trial and CP_trial cohorts did not show an association between negative response and insufficient coverage of HLA alleles by the epitopes in the set. Instead, this lack of specific T cells was explained by individual variation in the immune response. However, the limitation of our study is the absence of the individuals with later sampling time points (beyond 45 days after PCR). The rare responders in the HD-2021 group did not have an increased incidence of HLAs presenting the small number of cross-reactive peptides in the MHC-I set, and it is therefore most likely that these individuals were seronegative after asymptomatic infection.

Epitope immunogenicity, defined as the proportion of individuals with detectable response to the particular epitope among the total number of immunized HLA^+^ donors, varies significantly for different viral, cancer, or histocompatibility antigens (53–55). In particular, we have previously shown that, among 2 A*02:01-restricted SARS CoV-2 epitopes, _S1_YLQ is more immunogenic in comparison with _S8_RLQ, presumably due to higher T cell clonal diversity (7, 43). Several high-throughput screens have identified putative immunodominant epitopes (42, 49, 56, 57) and their HLA-restriction (Supplemental Table 1). However, the sizes of HLA^+^ groups in most of these screens were limited, preventing precise assessment of immunodominance and HLA restriction. By using a large number of immunized donors, we accurately measured immunogenicity of the epitopes and detected some additional restricting HLAs (e.g., K _S2_CY/A*11:01, _S6_QYI/A*23:01, and _N3_KTF/A*30:01). Several subdominant epitopes were not, to the best of our knowledge, assigned to the HLA before (e.g., _S4_FQP, _S5_GRL, _N7_SSP, _N5_QQQ, _S7_FCN, _O17_FLL, _M1_RYR, _S9_VRF, and _S29_LQT derived from TYVTQQLI), or at least not with statistical confirmation of their HLA restriction. Five precise epitopes with 2 or more detected responses were not reported in previous screens (42, 49, 56, 57) or were tested only within peptide pools and not individually (37). In contrast to MHC-I epitopes, MHC-II epitopes were mostly promiscuous in their HLA binding (Figure 3B). Most of the CD4^+^ response in our study was focused on non–S protein epitopes. Compared with the most comprehensive screen performed to date by Tarke et al. (49), we identified an additional 11 MHC-II epitopes and statistically confirmed HLA associations for 9 of them. Five precise epitopes were predicted from immunogenic peptides (50, 58); 6 others were annotated in IEDB epitopes of SARS-CoV-1. We also confirmed 2 MHC-I epitopes within MHC-II epitopes. Nevertheless, the epitopes, eliciting weak response and poor ex vivo T cell expansion, might be undetected by our assay, due to the limited sensitivity.

Comparison of natural immunity with postvaccination is critical for creating better vaccines. Indeed, convalescents, bearing a common HLA allele A*01:01, had a significantly higher response to MHC-I peptides than A*01:01^–^ individuals. Moreover, the response to the MHC I + II set in A*01:01 carriers exceeded the response to the full-length structural proteins, and this can probably be explained to a large extent by the presence of 7 confirmed A*01:01 epitopes — including 6 from nonstructural proteins, 5 of which were immunodominant — within the MHC I + II set. The notion that, in A*01:01 carriers, the majority of the CD8^+^ T cell response are focused on nonstructural proteins provides the rationale for designing vaccines that contain immunodominant peptides from proteins besides S. Moreover, it was recently shown that T cell response to early expressed nonstructural proteins may result in protection from the symptomatic infection (32). We did not observe an increased response for other HLAs, presenting multiple epitopes (e.g., 8 confirmed and 3 immunodominant epitopes for A*02:01).

Although multiple studies demonstrated equal T cell response of vaccinated and convalescent patients to the peptide pools spanning S protein (9–11, 13), the epitope repertoire recognized by these cohorts required further comparison and validation. We observed that T cells from Vac individuals recognized significantly more S protein–derived MHC-I epitopes than those from CP donors, while the total number of recognized MHC-I epitopes from any antigen was, unsurprisingly, higher in the latter group. Alongside the lack of a significant difference between these groups in the magnitude of the response to MHC-I peptides, this allows us to assume higher frequency — and, potentially, clonal diversity — of S protein–specific CD8^+^ T cells in vaccinated individuals, resulting from focusing of the response on a single antigen. However, this effect was not replicated in CD4^+^ T cells, and the number of recognized S protein–derived MHC-II epitopes per individual was significantly lower in the Vac cohort than in CP. It should be noted that multiple studies have suggested comparable CD8^+^ and CD4^+^ T cell responses at least 4 weeks after the first dose of vaccine (9–11, 13). Accordingly, we believe that the negligible PBMC response to recombinant S protein (Figure 2A) and low detection rate of S protein–specific CD4^+^ T cells after ex vivo expansion (Figure 3C and Supplemental Figure 4D) are associated with late sampling time (median 66 days after the first dose of vaccine). This suggests different dynamics of CD4^+^ T cells in Vac and CP cohorts or fading secretion of IFN-γ (used is a readout in our assays) by CD4^+^ T cells in vaccinee over the time. This is in line with the previous report in which CD4^+^ response peaked at 7 days, whereas CD8^+^ peaked at 42 days after boost (59). We believe that the lack of correlation between sampling time and response to S protein (Supplemental Figure 2D) is explained by the fact that, at the earliest sampling time in our study, the peripheral abundance of S protein–specific CD4^+^ T cells was already below the detection limit of the ELISpot assay.

Although there are several kits aimed at detecting SARS-CoV-2–specific T cells, we believe that the strategy of epitope selection is critical for discriminating preexisting cross-reactive immunity from specific immunity. Since we confirmed that the MHC I + II peptide set can accurately discriminate SARS-CoV-2–induced and cross-reactive immunity, we next characterized the HE cohort of individuals without clinical or laboratory signs of COVID-19 after prolonged contact with COVID-19 patients. Surprisingly, we saw virtually no response (1 of 37) to MHC I + II peptides, versus relatively frequent and robust responses to S, N, and M peptides, as well as significant responses to the cross-reactive _S26_IED and _N21_RWY peptides. The discordant responses in this group contrasted sharply with the CP cohort, in which responses to full-length antigens correlated strongly with responses to the MHC-II set (Supplemental Figure 2B). This is best explained by the activation of the preexisting cross-reactive T cells. The de novo recruitment of SARS-CoV-2–specific T cells from the naive compartment is unlikely, since, in contrast to CP cohort, it was not accompanied by the expansion of T cells specific to the panel of immunogenic SARS CoV-2 epitopes. Notably, we initially recruited 2 patients with a low-level IgM anti–S protein response who were subsequently excluded using a more sensitive commercial kit. These donors, however, demonstrated an easily detectable MHC I + II response (59.9 and 179.9 spots/1 × 10^6^ PBMC), suggesting that the reported peptide set could even be used to diagnose asymptomatic CP with a negligible humoral response. Based on these results, we believe that the described set of SARS-CoV-2 epitopes offers a sensitive and specific tool for the detection of COVID-19– or vaccination-induced (but not cross-reactive) T cell response.

## Methods

### Donors

#### The following describes donors for the non–clinical trial component of this study.

Healthy donors prepandemic (HD-2019, *n* = 52) included 29 PBMC samples and 23 cryobags obtained via mononuclear cell apheresis cryopreserved before December 2019. The CP samples (*n* = 51) included PBMCs from COVID-19 convalescents, collected and frozen in February–May 2020 (*n* = 43) or January–June 2021 (*n* = 8) within 20–70 days after the onset of symptoms. Assessments of individual epitope immunogenicity were performed on 48 of these samples. Vac PBMCs (*n* = 45) were collected and frozen 23–65 days after receiving the second dose of the Sputnik-V (Gam-COVID-Vac) vaccine from donors who had a negative antibody tests no later than 4 weeks before the first shot and had no self-reported COVID-like symptoms. ELISpot measurements were performed on 43 of these samples. The HE cohort (*n* = 37) of samples were from people who had close contact with a patient with active COVID-19 (same household or “red zone” medical workers with multiple negative RT-PCR tests) but who were, themselves, without COVID-19 symptoms and without detectable IgG and IgM anti–S protein antibodies.

The clinical trial of the Corona-T-test kit (NCT05165719) was conducted at Dmitry Rogachev National Medical Research Center of Pediatric Hematology, Oncology, and Immunology in Moscow, Russia, between July and September 2021. Donors were recruited by a separate center, DNKOM Laboratory (Moscow, Russia). The cohorts and inclusion criteria are listed in the Table 1.

Blood sampling and PBMC separation were performed with Vacutainer CPT Cell Preparation Tubes with sodium heparin for the separation of mononuclear cells from whole blood (BD Biosciences). Each PBMC sample was divided into 2 parts and used for measuring T cell response with the Corona-T-test kit and by flow cytometry with intracellular IFN-γ staining.

### PBMC isolation and HLA genotyping

In total, 30 mL of venous blood from healthy donors, vaccinated individuals, and recovered COVID-19 patients was collected into EDTA tubes and subjected to Ficoll density gradient centrifugation (400*g*, 30 minutes at room temperature). Isolated PBMCs were washed with PBS containing 2 mM EDTA twice, counted, cryopreserved in 7% DMSO and 93% heat-inactivated FBS (Capricorn Scientific), and stored in liquid nitrogen until used in the assays.

### HLA genotyping

For the Vac cohort, HLA genotyping was performed with the HLA-Expert kit (DNA-Technology LLC) through the amplification of exons 2 and 3 of the HLA-A/B/C genes and exon 2 of the HLA-DRB1/3/4/5/DQB1/DPB1 genes. Prepared libraries were run on an Illumina MiSeq sequencer using a standard flow-cell with 2 × 250 paired-end sequencing. Reads were analyzed using HLA-Expert Software (DNA-Technology LLC) and the IPD-IMGT/HLA database 3.41.0 (10.1093/nar/gkz950).

For CP_trial, Vac_trial, HD-2021, and CP donors, HLA-genotyping was performed using the One Lambda ALLType kit (Thermo Fisher Scientific), which uses multiplex PCR to amplify full HLA-A/B/C gene sequences, and from exon 2 to the 3′ UTR of the HLA-DRB1/3/4/5/DQB1 genes. Prepared libraries were run on an Illumina MiSeq sequencer using a standard flow-cell with 2 × 150 paired-end sequencing. Reads were analyzed using One Lambda HLA TypeStream Visual Software (TSV), version 2.0.0.27232, and the IPD-IMGT/HLA database 3.39.0.0. Two donors (p1305, p1329) were HLA genotyped by Sanger sequencing for loci HLA-A, HLA-B, HLA-C, DRB1, and DQB1, using Protrans S4 and Protrans S3 reagents. The PCR products were prepared for sequencing with BigDye Terminator v1.1 (Thermo Fisher Scientific). Capillary electrophoresis was performed on a Genetic Analyzer Nanophore05.

### IFN-γ ELISpot for nonclinical testing

Cells from cryotubes or blood cryobags were thawed, rested 4–24 hours in CTL test media, counted, and plated in human IFN-γ ELISpot plates (CTL) at 3 × 10^5^ PBMCs per well and incubated with different sets of antigens: 1 μM peptides MHC-I, 1 μM MHC-II cross^+^, 1 μM MHC-II cross^–^, 10 μg/mL full-length S protein, or 1 μg/mL commercial peptide pools covering S, N, and M proteins (Miltenyi Biotec). S protein–derived peptides consisted of a combination of PepTivator SARS-CoV-2 Prot_S, PepTivator SARS-CoV-2 Prot_S1, and PepTivator SARS-CoV-2 Prot_S+. Full-length recombinant S protein was produced as described earlier (7). For the positive control, we used 40 ng/mL PMA (P8139-1MG, MilliporeSigma) and 400 ng/mL calcium ionophore (C7522-1MG, MilliporeSigma), with 0.02% isopropanol plus 0.02% DMSO as a negative control. After 18–20 hours of incubation at 37°C, 5% CO_2_, the plates were developed according to the manufacturer’s guidelines. Spots were counted using the CTL ImmunoSpot Analyzer. T cell responses were considered positive when more than 7 spots (mean of duplicates) were detected after subtracting the negative control. Samples with > 17 spots in the negative control or < 50 spots in the positive control were excluded from the analysis. Cut-off of 7 spots was preevaluated on the results of 20 negative control wells from different donors. It was calculated as max_value + 3SD = 6.03. All ELISpot data are presented as number of spots per 1 × 10^6^ cells, and cutoff is adjusted accordingly.

### T cell expansion

PBMCs from cryotubes or blood cryobags were thawed, rested 4–24 hours in CTL test medium, counted, and used for rapid in vitro expansion. In total 2 × 10^6^ to 4 × 10^6^ cells per well were plated in 24-well plates in RPMI 1640 culture medium supplemented with 10% normal human A/B serum, 1 mM sodium pyruvate, 2 mM GlutaMAX Supplement (Thermo Fisher Scientific), 25 ng/mL IL-7, 40 ng/mL IL-15, and 50 ng/mL IL-2 (Miltenyi Biotec) at a volume of 1 mL/well. The initial full set of peptides (final concentration of each = 10 μM) were added on day 0. On day 3, 1 mL of supplemented medium without peptides was added to each well (final volume 2 mL). Half of the medium was replaced on days 5, 7, and 10. On days 10 and 11, an aliquot of cell suspension was used for anti–IFN-γ ELISA with pooled peptides and individual peptides, respectively. On day 13, cells were sampled for flow cytometry analysis. In order to maintain detectable IFN-γ secretion, a quarter of the medium was replaced with supplemented medium on days 11 and 13.

### Cell stimulation with peptide pools

After 10 days of expansion, an aliquot of cell culture was washed twice in 1.5 mL of PBS and was then transferred to AIM-V medium (Thermo Fisher Scientific), plated at 1 × 10^5^ cells per well in 96-well plates, and incubated overnight (12–16 hours) with the peptide pools (ELISA pools, see above). In total, 0.04% DMSO and 0.04% isopropanol were used as negative control, with 40 ng/mL PMA and 400 ng/mL calcium ionophore as positive control. On day 11, the culture medium was collected and tested for IFN-γ as described below. On days 11–12, we stimulated the cells as described above individually with each peptide (2 μM) from the pools. Only peptides predicted to bind to each individual’s HLA were tested. Finally, on day 13, we stimulated the MHC-II peptide–expanded cells with the MHC-II peptides that generated a positive response with ELISA for flow cytometry experiments.

### Anti–IFN-γ ELISA

Ninety-six–well high–protein binding ELISA plates (Shanghai Meikang Biological Project, KH-M-02) were coated with 100 μL per well of 0.01 mg/mL anti–IFN-γ antibody (Hytest, clone GF1) in 100 mM bicarbonate/carbonate (pH 9.6). After 14 hours, the plates were washed once with 250 mL PBS + 0.1% Tween 20 (PBST) and blocked with 100 mL of 1% hydrolyzed casein (XEMA) in PBS for 3 hours at room temperature, dried, and stored sealed at 4°C.

Culture plates were centrifuged for 3 minutes at 700*g*, and 100 μL of the medium was transferred to the ELISA plates. Plates were incubated for 1.5 hours at 37°C on a rocking platform. The plates were washed 3 times with PBST, and then 100 μL of 0.1 μg/mL biotinylated anti–IFN-γ antibody (R&D Systems) was added and incubated for 1 hour at 37°C on a rocking platform. The plates were washed 3 times with PBST, and then 100 μL of Streptavidin-HRP (XEMA) was added and incubated for 0.5 hours at 37°C on a rocking platform. Finally, the plates were washed 5 times with PBST, and 100 μL of 3,3’,5,5’-tetramethylbenzidine substrate (XEMA) was added to each well. Fifteen minutes later, 100 μL of 1M H_2_SO_4_ was added as a stop solution, and the OD was measured at 450 nm on a MultiScan FC (Thermo Fisher Scientific) instrument. Each plate included standards corresponding to 0, 15, 120, and 7700 pg/mL of IFN-γ to control the sensitivity and linearity.

Test wells with peptides where the ratio OD_450_test_well_/OD_450_negative_ _control_ ≥ 1.25 and the difference OD_450_test_well_ – OD_450_negative_ _control_ ≥ 0.08 were considered positive. Peptides with a ratio between 1.25 and 1.5 were tested again up to 3 times as biological replicates to ensure the accuracy of their response, and peptides with 2 or 3 positive results were considered positive. All other peptides were considered negative. If ELISA results conflicted with flow cytometry data (for MHC-II peptides), the peptide was considered nonimmunogenic.

### Flow cytometry (for nonclinical testing)

After 13 days of expansion, an aliquot of cell suspension was washed twice in 1.5 mL of PBS; it was then resuspended in AIM-V medium with 1.0 μg/mL brefeldin A (GolgiPlug, BD Biosciences). Cells were plated at 1 × 10^5^ per well in a 96-well polypropylene V-bottom plate and incubated at 37°C for about 5 hours with MHC-II peptides that were identified as positive in the previous ELISA assay. In total, 0.04% DMSO and 0.04% isopropanol were used as a negative control, and 40 ng/mL PMA and 400 ng/mL calcium ionophore were used as positive control. Stimulation was stopped by washing with PBS plus 0.5% BSA (Sigma-Aldrich) and 2 mM EDTA. Surface staining was performed for 10 minutes with Alexa Fluor 750 NHS Ester (catalog A37575; Thermo Fisher Scientific) in 100 μL PBS at room temperature, followed by 2 washes with 10% FBS diluted with PBS and staining with anti–CD3-FITC (SK7; catalog 345764; BD Biosciences), anti–CD4-PE (SK3; catalog 345769; BD Biosciences), and anti–CD8-APC (SK-1; catalog 345775; BD Biosciences) for 20 minutes at 4°C. Fixation and permeabilization were performed with BD Cytofix/Cytoperm fixation and permeabilization solution (catalog 555028; BD Biosciences), according to the manufacturer’s protocol, followed by IFN-γ–BV421 (B27; catalog 562988; BD Biosciences) staining for 20 minutes at 4°C. All samples were analyzed with a MACSQuant Analyzer 10 (Miltenyi Biotec). Instrument performance was monitored prior to every measurement with MACSQuant Calibration Beads (Miltenyi Biotec). The acquired data were processed by FlowJo software (version 10.6.2., Tree Star Inc.). The percentage of IFN-γ^+^ cells was calculated in the CD3^+^CD8^+^ and CD3^+^CD4^+^ gate. The difference or ratio (fold-change) of the percentage of IFN-γ^+^ cells incubated with peptide and with negative control was calculated. Peptides with a ratio > 2 were considered positive for CD4^+^ recognition, and those with a ratio > 3 were considered positive for CD8^+^ recognition due to a higher background percentage of CD8^+^IFN-γ^+^ cells. Wells containing < 5000 CD8^+^ cells were not analyzed for the CD8^+^ response. In cases of conflicting results between ELISA and flow cytometry, the peptide was considered negative.

### Anti–SARS-CoV-2 ELISA

ELISA kits for the detection of anti–RBD IgG (K153, National Research Center for Hematology) and SARS-CoV-2 IgМ-EIA-BEST (D-5502, Vector Best) for the detection of IgM antibodies to full-length S protein were used according to the manufacturers’ instructions.

### Assessing epitope homology and mutations in VOC

To assemble the control peptide pool, IEDB was queried for epitopes with positive MHC binding and a minimum of 2 positive T cell assays using “Severe acute respiratory syndrome-related coronavirus” as “Organism” on October 11, 2021. MHC-I and MHC-II peptides used in this study were excluded from the IEDB epitope pool. Alignments were performed using best global-local alignment by the “pairwiseAlignment” (bioPython) function for 4 ORFs shared by all 5 strains — orf1ab, S, N, and M — allowing amino acid substitutions with similar biochemical properties (1, 2) and low penalties for gap opening and extension (–0.5). The segments of the alignments corresponding to the given SARS-CoV-2 peptide were further aligned with high gap penalties (–10/–1) followed by calculation of the similarity score (60, 61) and identity score.

Mutation analysis was performed using data extracted from t-cov.hse.ru (62) (accessed December 15, 2021) and included 12 VOC strains. The HLA binding of the mutated peptide compared with the reference peptide was not taken into consideration.

### HLA and epitope selection

We selected the HLA list based on the most-presented HLA among the CP cohort: A*01:01; A*02:01; A*03:01; A*11:01; A*23:01; A*24:02; A*25:01; A*30:01; A*32:01; B*07:02; B*08:01; B*13:02; B*15:01; B*18:01; B*27:05; B*35:01; B*38:01; B*40:01; B*44:02; B*44:03; B*51:01; C*01:02; C*03:04; C*04:01; C*05:01; C*06:02; C*07:01; C*07:02; C*12:03; C*15:02; DRB1*01:01; DRB1*07:01; DRB1*11:01; DRB1*11:04; DRB1*13:01; DRB3*01:01; DRB3*02:02; DRB4*01:01; DQB1*02:01; DQB1*03:01; DQB1*03:02; DQB1*05:01; and DQB1*06:03.

#### MHC-I peptides.

In addition to 2 epitopes from Shomuradova et al. (7) and 25 minimally/non-cross-reactive epitopes from the publications listed in Figure 1A, we selected 67 epitopes from Snyder et al. (37). We preferred individual epitopes instead of peptides within a peptide pool and balanced predicted HLA coverage, minimal cross-reactivity (defined as the number of the detected responses to a specific epitope/peptide group within nonnaive T cell expansions from healthy donors), and higher immunogenicity (based on the number of convalescents with detected response to a specific epitope/peptide group). We predicted HLA binding of the selected MHC-I peptides, using NetMHCPan4.1 (63) with standard thresholds; strong and weak binders were considered as potential epitopes for a given HLA allele. The references for each protein are given in Supplemental Table 1.

#### MHC-II peptides.

MHC-II peptides were minimally cross-reactive and immunogenic peptides from the publications listed in Figure 1A. Thereafter, we searched for the exact MHC-II epitopes from those peptides and predicted their binding to the selected HLA-II epitopes assigned to SARS-CoV-1 in IEDB and 4 peptides with predicted high binding promiscuity (>7 HLA-II alleles). HLA binding was predicted using both NetMHCIIpan 3.2 (64) and Neon MHC2 (65) for MHC-II peptides using standard thresholds. Strong binders, weak binders, or peptides with discordant NetMHCIIpan and Neon MHC2 predictions were considered as potential binders and were tested in donors bearing those HLAs. The references for each protein are given in Supplemental Table 1.

### Peptides and mixes

Peptides (at least 95% purity) were synthesized either by Peptide 2.0 Inc. or by the Shemyakin-Ovchinnikov Institute of Bioorganic Chemistry RAS. Peptides containing Cys and/or Met were diluted in 50% isopropanol in PBS at concentrations of up to 10–25 mM. Other peptides were diluted in DMSO (MilliporeSigma) at up to 30–40 mM. When peptide pools were prepared, peptides containing Cys/Met were not mixed with peptides in DMSO. All individual peptides and mixes were aliquoted for single use and stored at –80°C.

For identification of cross-reactive peptides, matrix pools were prepared (20 matrices for MHC-I and 11 for MHC-II), with each peptide present only in 2 pools. IFN-γ production in wells containing both pools indicated the specificity of the corresponding peptide (Supplemental Figure 1F). We used the following mixes in our work (each presented by 2 submixes, diluted in DMSO or isopropanol):

For the ELISpot assay (final concentration in medium = 1 μM): (a) MHC-I peptides, containing all MHC-I peptides; (b) MHC-II peptides (cross^+^) for all MHC-II peptides; (c) MHC-II peptides cross^–^ for MHC-II peptides without _N21_RWY and _S26_IED; (d) MHC I + II_IVD for premixed, aliquoted, and lyophilized set of 115 peptides without _N21_RWY, _S26_IED, and _M9_SEL (the latter was excluded due to its physicochemical properties hindering synthesis); and (e) matrix mixes (20 with MHC-I peptides and 11 with MHC-II peptides).

For T cell expansion (final concentration in medium = 10 μM), the full set of peptides was split into 5 standard mixes (expansion pools; Supplemental Table 1).

For T cell expansion (final concentration in medium = 10 μM), the full set of peptides was split into 5 standard mixes (expansion pools). For the first step of epitope identification in anti–IFN-γ ELISA, we used 26 standard mixes containing 3–5 peptides (ELISA pools).

### Measuring T cell response with Corona-T-test, for the clinical trial

Corona-T-test is a single-color enzymatic ELISpot kit for IFN-γ detection produced by the National Research Center for Hematology. After washing cells with sterile RPMI 1640 media twice, cells were counted in a hemocytometer (Sysmex XS-1000i, Sysmex Corporation) and resuspended in serum-free AIM-V + AlbuMAX BSA (Thermo Fisher Scientific) medium to a concentration of 6 × 10^6^/mL; then, 3 × 10^5^ cells were loaded per well. We used 4 wells (final volume 150 μL) for each PBMC sample; negative control was stimulated with AIM-V medium, SARS-CoV-2 antigen–induced stimulation in duplicate (MHC I + II_IVD, 1 μM/mL), and positive control with PHA (10 μg/mL). Plates were incubated 16–18 hours at 37°C, with 5% CO_2_. The next day, the plates were developed according to the manufacturer’s guidelines, with spots counted using the automated ImmunoSpot Series 5 UV Analyzer (CTL). Results were considered valid if the number of spots was < 10/well in negative controls and > 100/well in positive controls. Nonspecific activation in negative controls was subtracted from the average of the 2 stimulated sample wells. Responses with < 4 spots were considered negative and > 6.5 spots were considered positive. Gray zone samples with 4–6.5 spots were considered inconclusive, requiring repeated testing, and such samples (*n* = 6) were excluded from the accuracy analysis. Data are presented as number of spots per 1 × 10^6^ cells; cutoff and gray zone are adjusted accordingly.

### Flow cytometry, for the clinical trial

PBMCs were separated as described previously and rested overnight at 7.5 × 10^6^ cells/mL in AIM-V + AlbuMAX (BSA) medium at 37°C with 5% CO_2_. The next day, 200 μL of cell suspension was added to each well of a 96-well polypropylene V-bottom plate. All samples received 0.25 μL of brefeldin A at the beginning of incubation. For stimulation, 1 μM MHC I + II_IVD peptides were added to the test wells, 1.5 μg/mL PHA (Capricorn Scientific GmbH) was used as a positive control, and nonstimulated cultures were used as negative control. The cells were incubated for 4 hours at 37°C, 5% CO_2_ and then transferred into 12 × 75 mm plastic tubes at 1.5 × 10^6^ cells per tube and washed with 2 mL MACS PBS/EDTA Buffer without Ca^2+^ or Mg^2+^ (Miltenyi Biotec) with 0.5% heat-inactivated MACS BSA (Miltenyi Biotec; PBS/BSA/EDTA). The cells were centrifuged at 350*g* for 5 minutes at room temperature and then stained. Cell surface staining of T cells was done in 0.1 mL PBS/BSA/EDTA for 15 minutes with FITC-conjugated anti-CD3 (SK7; catalog 345764; BD Biosciences), PE-Cy7 anti-CD8 (SK1, catalog 335822; BD Biosciences), VioGreen anti-CD4 (REA623, catalog 130-113-230, Miltenyi Biotec), and 7–amino-actinomycin D (7-AAD, catalog 130-111-568, Miltenyi Biotec) in the dark at room temperature. Fixation and permeabilization were performed with BD Cytofix/Cytoperm according to the manufacturer’s protocol, and intracellular staining was carried out for 30 minutes in the dark at room temperature with APC anti–IFN-γ (B27, catalog 554702, BD Biosciences). Cells were analyzed on a CytoFLEX (Beckman Coulter) flow cytometer. Instrument performance was monitored daily with CytoFLEX Daily QC Fluorospheres (Beckman Coulter). Individual populations were gated according to forward scatter, side scatter, and specific markers, and the data were subsequently analyzed with CytExpert software (Beckman Coulter). Dot plots representing anti-CD3 versus anti–IFN-γ were carried out to establish CD3^+^IFN-γ^bright^ lymphocyte gates. Identical dot plots were generated for CD8^+^IFN-γ^bright^ and CD4^+^IFN-γ^bright^ cells. Typically, 300,000 events were acquired in the gating CD3^+^ region. Nonspecific activation in unstimulated controls was subtracted from stimulated samples to account for specific activation.

### Statistics

All data analysis was performed using GraphPad Prism 9, Python 3.2, FlowJo 10, and CytExpert software. The statistical test used is indicated in each figure legend. For detection of the statistical association between HLA alleles and MHC-II epitopes or non–S protein–derived MHC-I epitopes, we used only the CP cohort; for HLA-I alleles and S protein–derived MHC-I epitopes, we used both CP and Vac cohorts. The number of the epitope responders among HLA carriers and the number of responders among individuals without this HLA were compared with Fisher’s exact test, with *P* < 0.05 considered significant.

### Study approval

The study was approved by the National Research Center for Hematology ethical committee. All donors signed the informed consent form approved by the National Research Center for Hematology ethical committee before enrollment. All patients were insured during the clinical trial.

## Author contributions

Conceptualization was contributed by GAE and A Titov. Methodology was contributed by GAE, A Titov, RS, AK, and DBM. Data analysis was contributed by A Titov, RS, AK, and DBM. Investigation, A Titov, RS, OVS, YVS, AVM, VAV, DEP, RVN, and EYO. Resources were contributed by KVZ, SAS, NTS, DVD, EK, MS, SN, A Tonevitsky, and AVE. Writing of the original draft was contributed by A Titov. Review and editing were contributed by GAE, A Titov, RS, AK, VRG, and SAS. Project administration was contributed by GAE, VRG, MM, and AVE. Funding acquisition was contributed by GAE.

## Supplementary Material

Supplemental data

Supplemental tables 1-3

## Figures and Tables

**Table 1:**
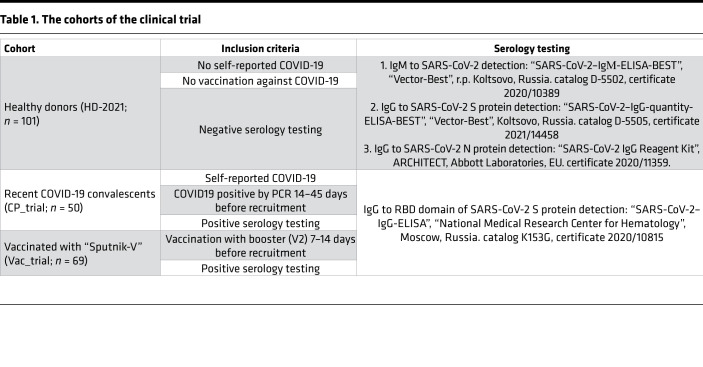
The cohorts of the clinical trial

## Version 1 04/07/2022

In-Press Preview

## Version 2 05/09/2022

Electronic publication
