# Supplementary material for: Immunogenic epitope panel for accurate detection of non-cross-reactive T cell response to SARS-CoV-2
Source: JCI Insight. 2022 May 9;7(9):e157699. doi: 10.1172/jci.insight.157699 (PMC9090254; doi:10.1172/jci.insight.157699)
Supplement: Supplemental data [file jciinsight-7-157699-s256.pdf]

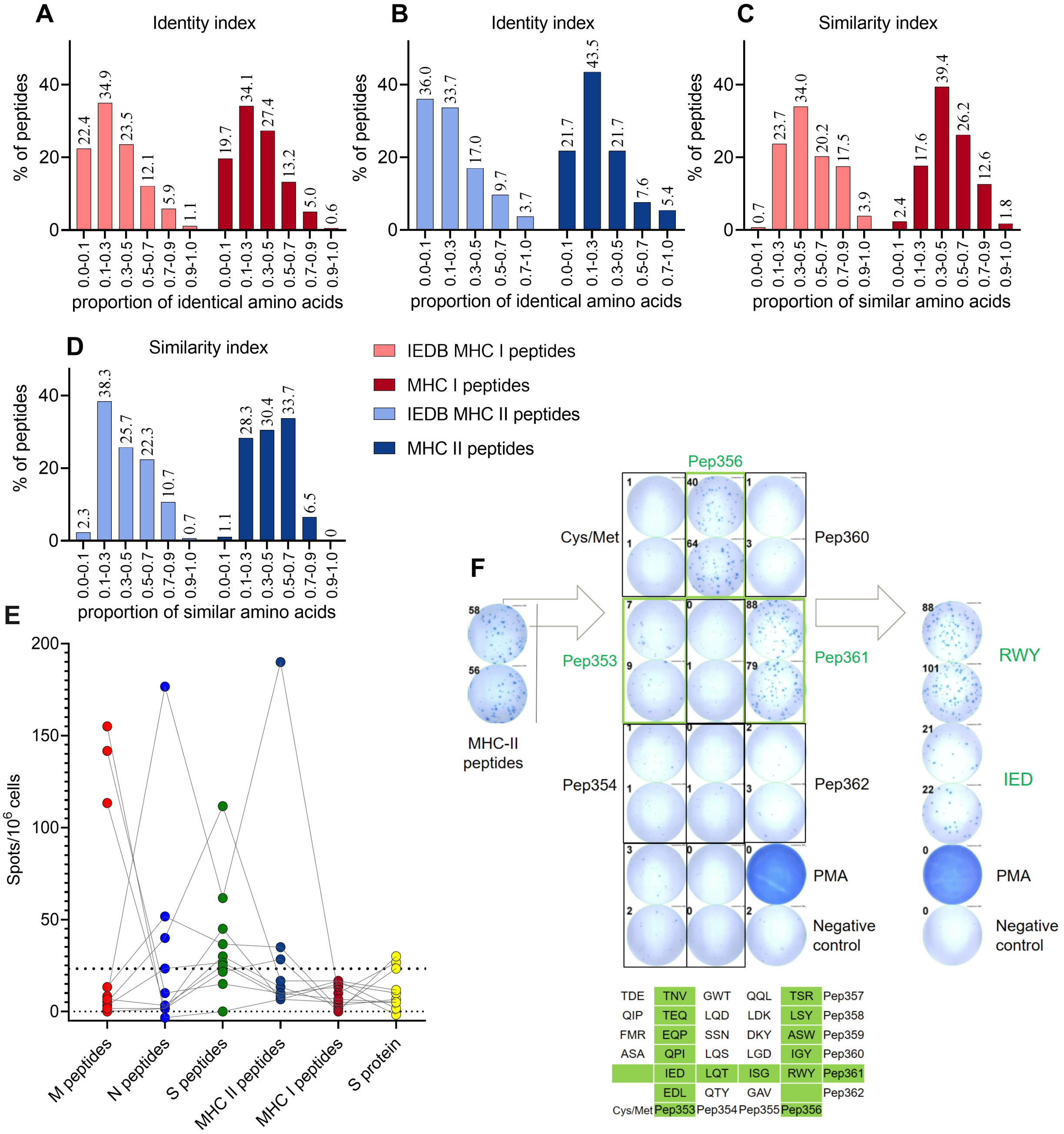

TDE	TNV	GWT	QQL	TSR	Pep357
QIP	TEQ	LQD	LDK	LSY	Pep358
FMR	EQP	SSN	DKY	ASW	Pep359
ASA	QPI	LQS	LGD	IGY	Pep360
	IED	LQT	ISG	RWY	Pep361
	EDL	QTY	GAV		Pep362
Cys/Met	Pep353	Pep354	Pep355	Pep356	

Figure S1. (A–D). Identity (A and B) and similarity (C and D) of the MHC-I (A and C) and MHC-II (B and D) peptides, aligned to common cold coronavirus (229E, NL63, HKU1, OC43) proteins. Peptides from IEDB were used as a control. (E) Response to the indicated antigens among healthy donors (grouped for each donor). Responders to at least one antigen are shown. Dots show the mean of two duplicates with negative control subtracted. Threshold for positive result is indicated by the dotted line. (F) The strategy for identifying cross-reactive MHC-II epitopes. This particular donor responded to MHC-II peptides. Identification was performed using matrix pulls; the peptide composition of those pools is indicated in the table at lower left. Green marks matrix pools that evoked a response. When we tested the same donors' peripheral blood mononuclear cells (PBMCs) against the individually-identified peptides (right) we observed a matching response.

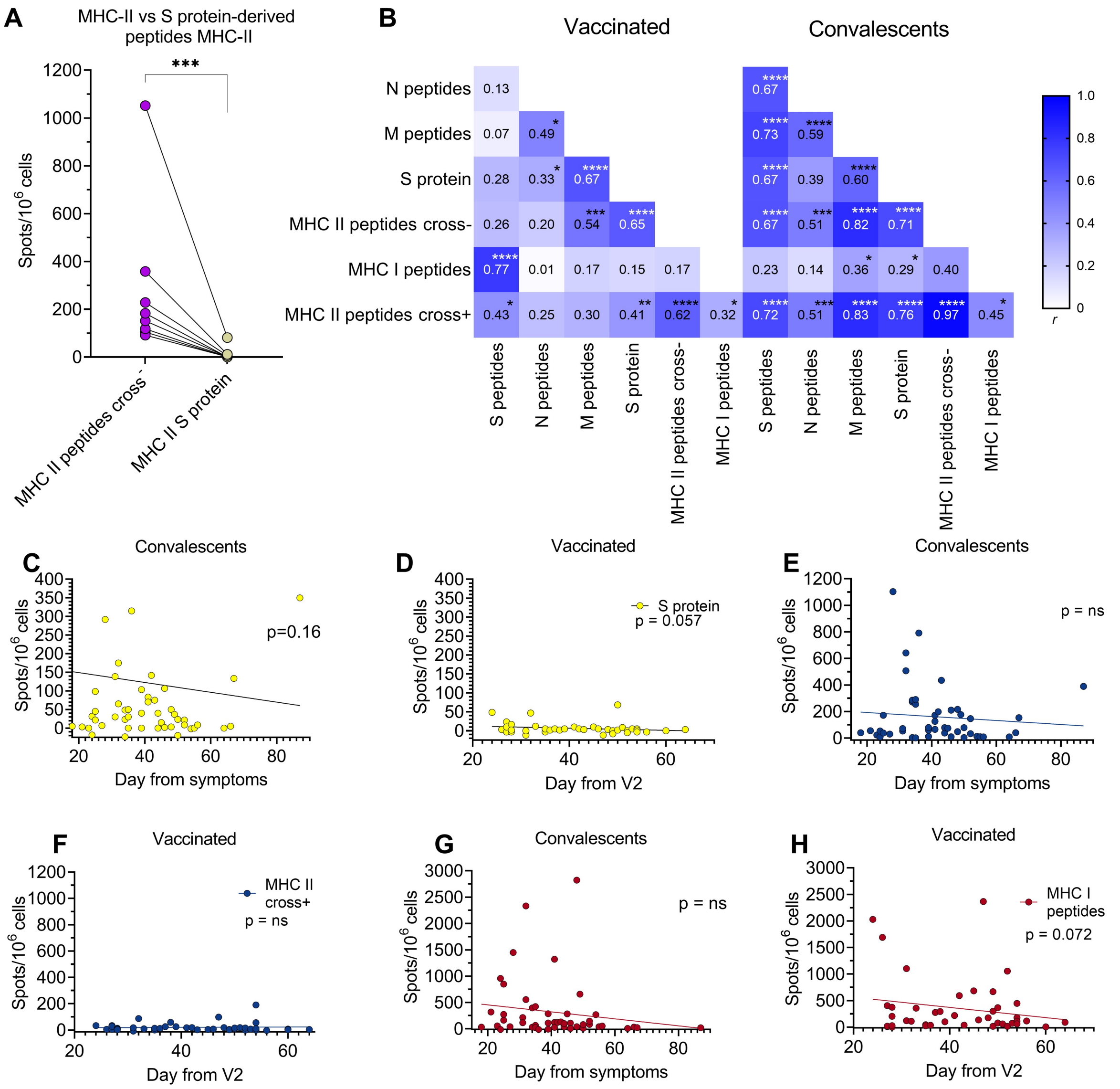

Figure S2. (A) Difference in response to MHC-II peptides versus S protein-derived MHC-II peptides (Wilcoxon test, p=0.002). (B) Correlation between responses to different antigens in Vac and CP (Spearman correlation, *p < 0.05, **p < 0.01, ***p < 0.001, ****p < 0.0001). (C–H) Influence on time elapsed from symptoms or boost vaccination (V2) on the magnitude of response to recombinant S protein in CP (C) and Vac (D), MHC-II peptides in CP (E) and Vac (F), and MHC-I peptides in CP (G) and Vac (H) (linear regression).

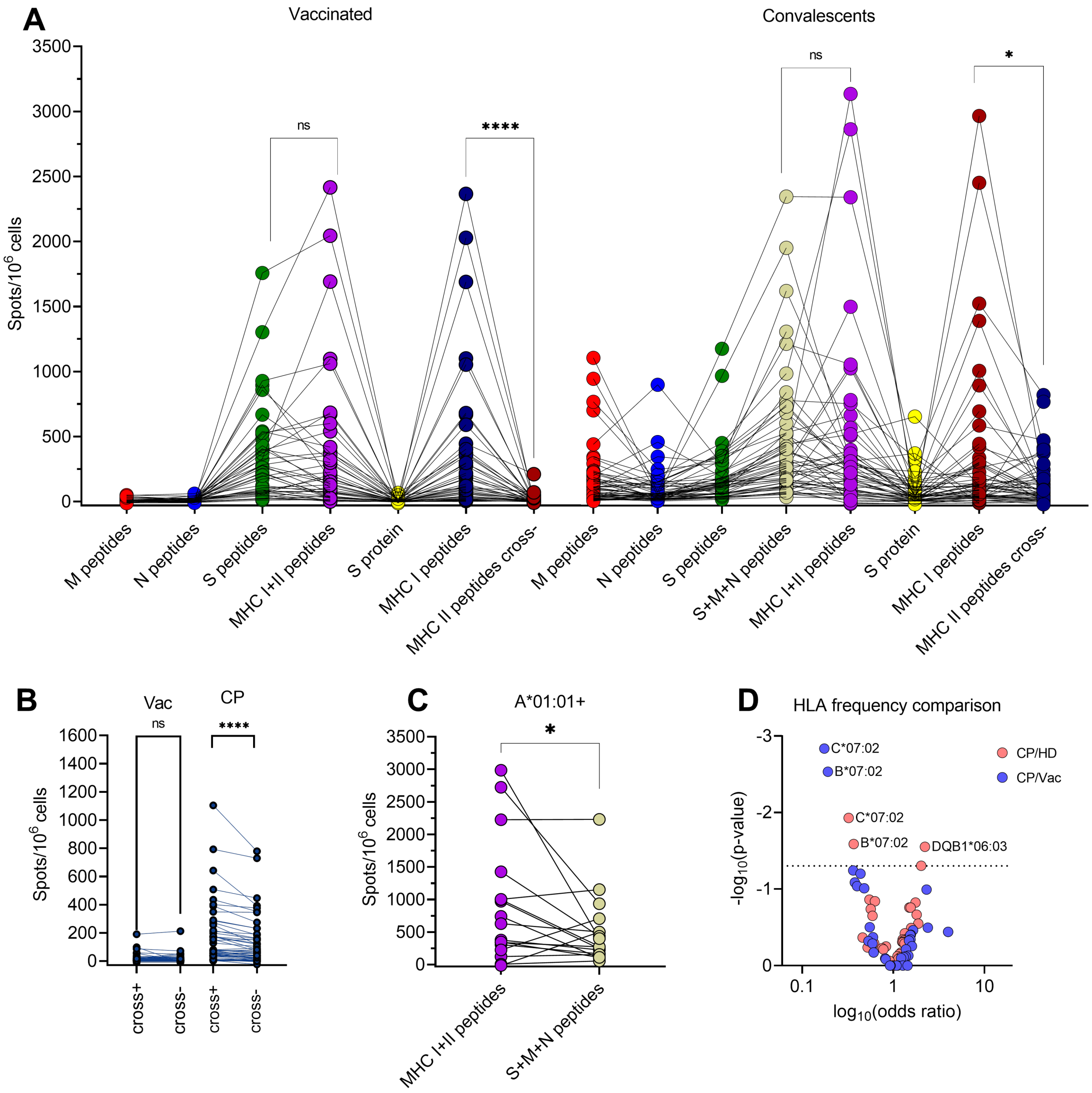

Figure S3. (A) Response to antigens and peptide sets grouped by individuals. (B) Difference in responses to MHC-II peptides before and after exclusion of two cross-reactive peptides (Wilcoxon test, ****p<0.0001, *p=0.038) (C) Response to antigens among A*01:01⁺ convalescents (Wilcoxon test, p=0.046). (D) Volcano plot shows the difference between HLA allele frequencies in groups. The x-axis denotes the decimal logarithm of the odds ratio of the given allele carriers and non-carriers between different cohorts (HD - bone marrow donors, n=2210). Y-axis denotes the negative decimal logarithm of the p-value. P-value = 0.05 is depicted by the dotted line (Fisher exact test, statistically significant values are annotated).

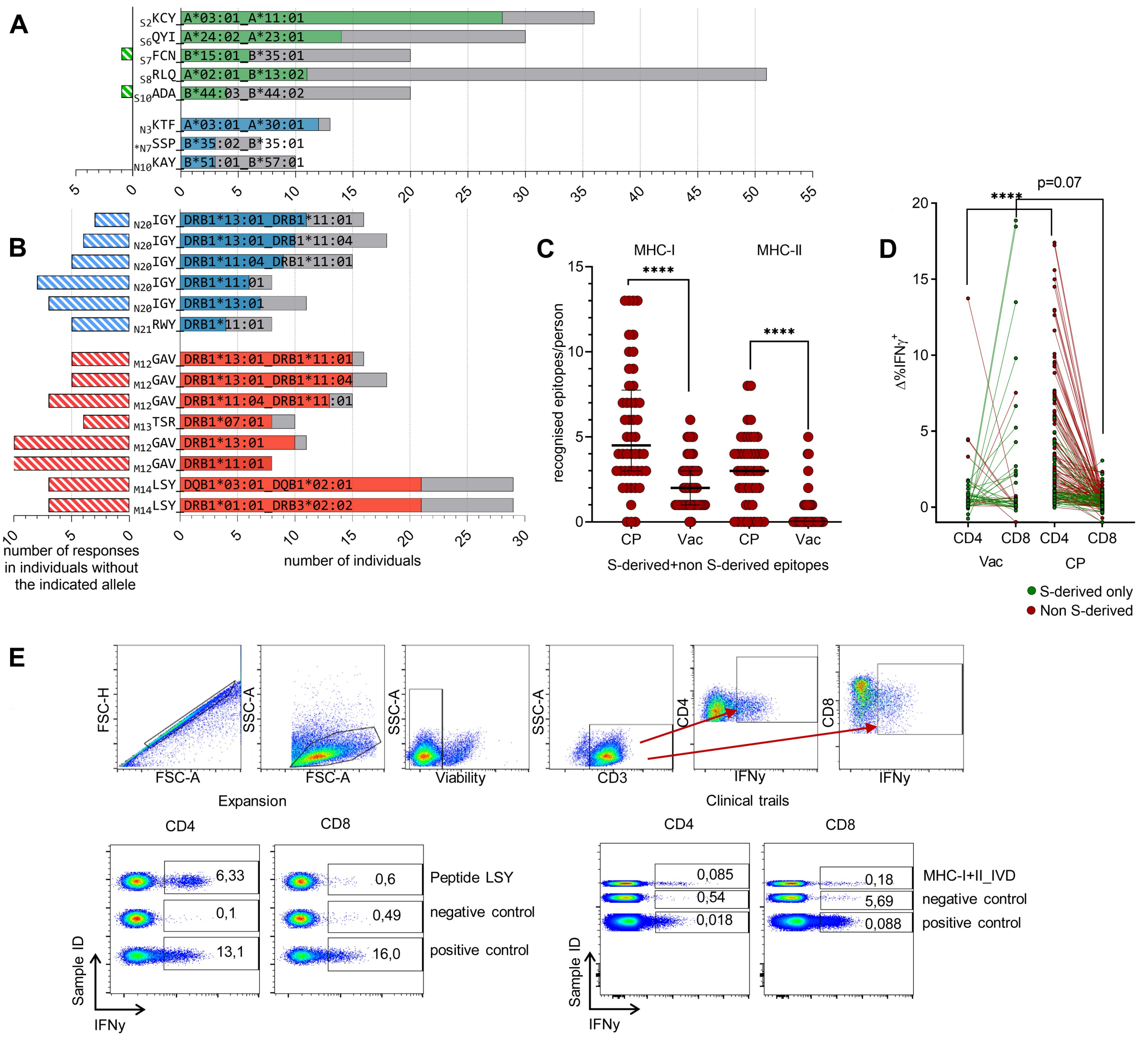

Figure S4. (A, B) Number of responses to each peptide in individuals with (right) or without (left) the indicated (A) HLA-I or (B) -II alleles. Data are presented as in **Figure 3A, B**. (C) Number of any protein-derived MHC-I and MHC-II epitopes recognized per individual for the CP and Vac cohorts. (D) Flow cytometric analysis of the phenotype of T cells responding to MHC-II peptides after *ex vivo* expansion. Plot shows the difference in the % of $CD4^{+}$ or $CD8^{+}$ $IFN\gamma^{+}$ T cells between peptide-stimulated cells and negative controls. Mann-Whitney test, ****p<0.0001 (E) Gating strategy for expansion **(Fig. S4D)** and clinical trial **(Fig. S6A-B)** flow cytometry data and representative cytometry plots for expansion (bottom left, response to LSY peptide, negative and positive control) and clinical trial (bottom right, response to the MHC-I+II_IVD mix, negative and positive control).

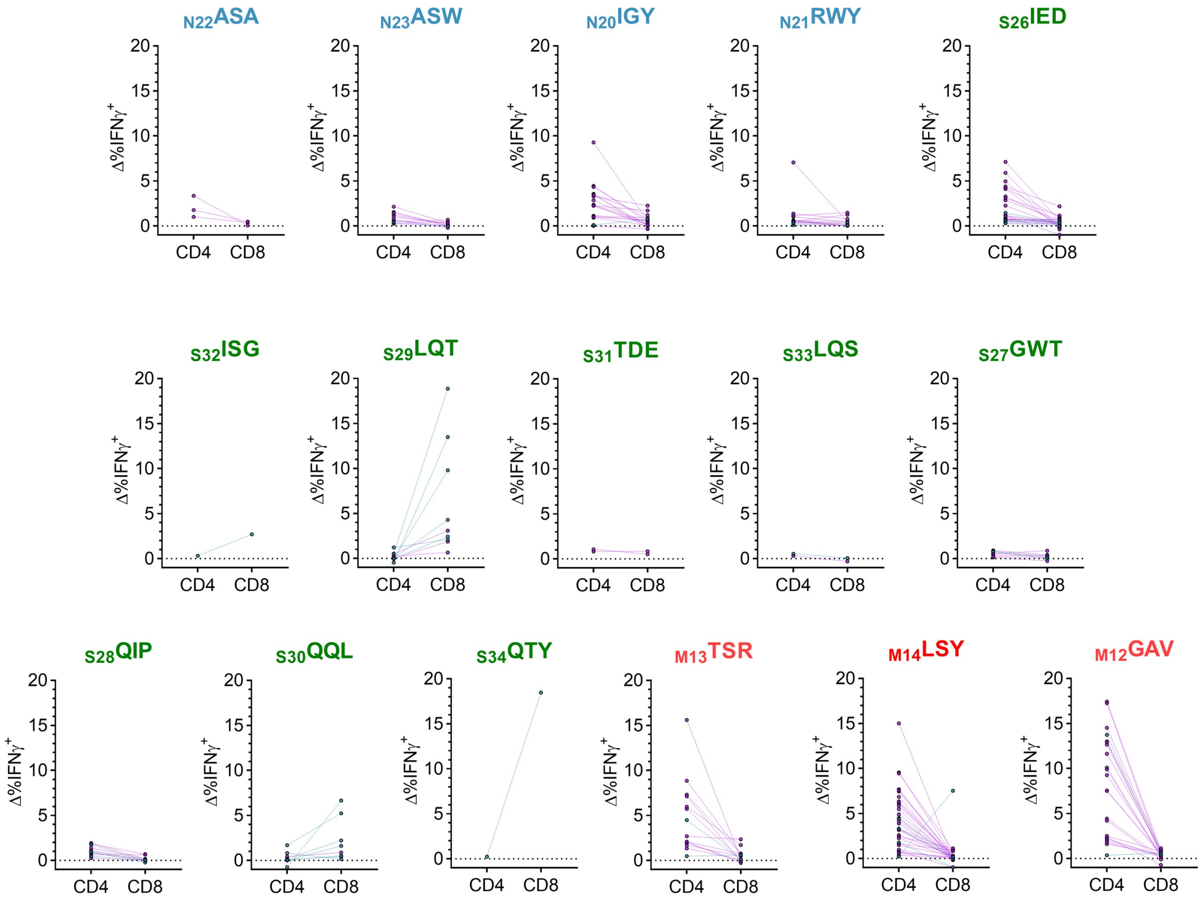

Figure S5. Flow cytometric analysis of the phenotype of *ex-vivo* expanded T cells responding to MHC-II peptides for each peptide (antigens labeled green for S, red for M, and blue for N) in Vac (blue) and CP donors (rose). Plots show differences in the % of CD4 or CD8 IFNɣ+ T cells after *ex-vivo* expansion between peptide-stimulated cells and negative controls.

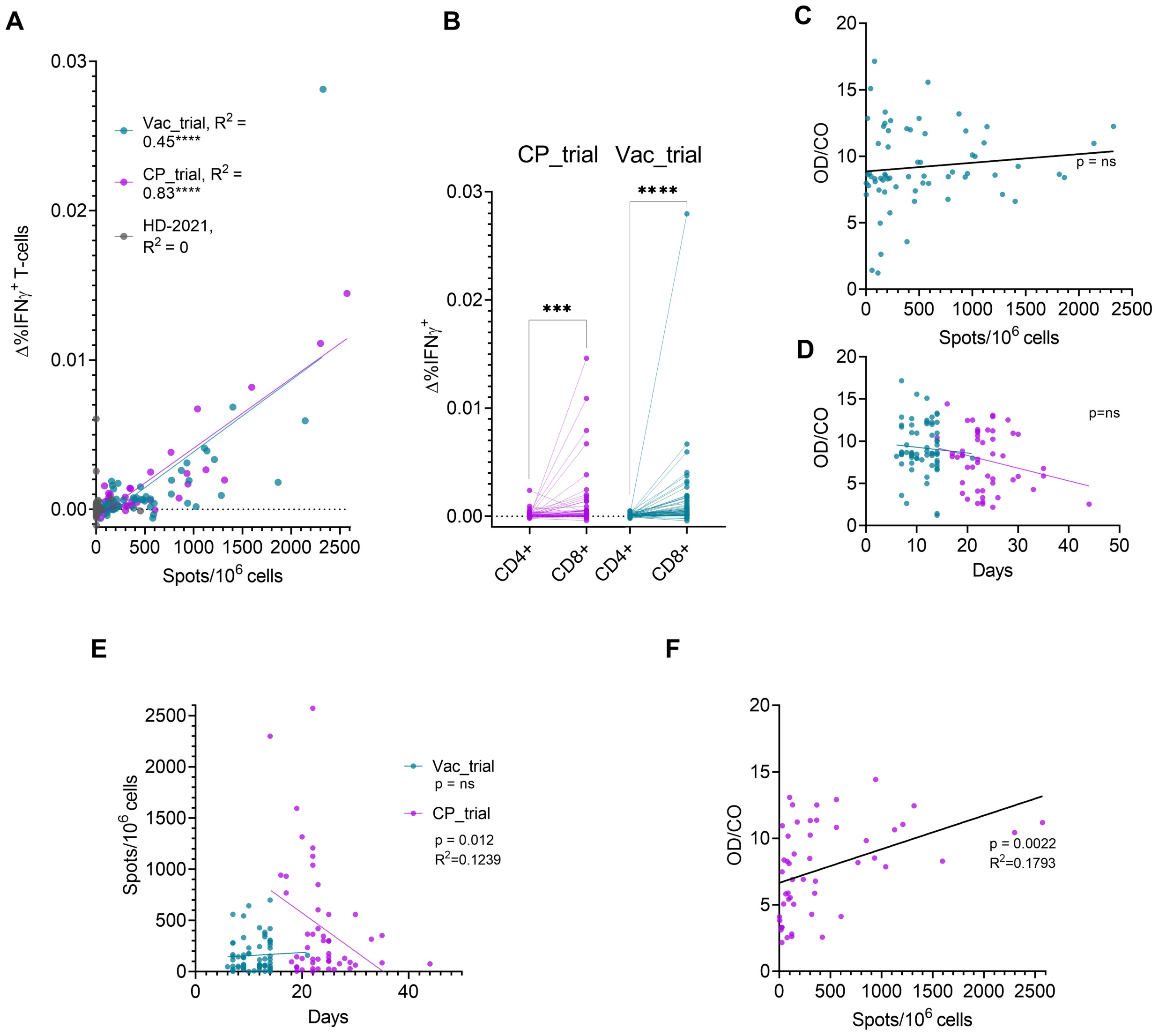

Figure S6. (A) Scatter plot shows response of Vac_trial (n = 60), CP_trial (n = 48), and HD-2021 (n = 88) cohorts (only donors with available flow cytometry data are shown) as measured by Corona-T-test (number of spots; x-axis) versus flow cytometry (sum of $\%CD4^+$ $IFNγ^+$ and $\%CD8^+$ $IFNγ^+$ after subtracting background; y-axis). Linear regression, F-statistic, ****$p < 0.0001$. (B) Impact of $CD4^+$ and $CD8^+$ on the total response. Plot shows the difference between the peptide-stimulated PBMC and negative control in $IFNγ^+$ cells gated as $CD4^+$ or $CD8^+$ cells. Wilcoxon test, ***$p = 0.0009$, ****$p < 0.0001$ (C) Correlation between Corona-T-test response and time since V2 (boost vaccination) in Vac_trial individuals. (D, E) Correlation between time since V2 in Vac_trial or disease onset in CP-trial and (D) humoral or (E) T cell response. (F) Correlation between Corona-T-test response and time since disease onset in convalescents. OD/CO represent the ratio of OD_{450} of the sample to the test cut-off.

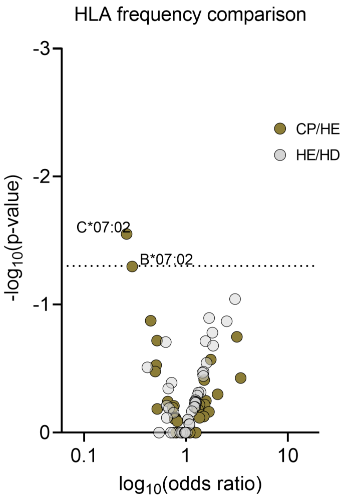

Figure S7. Volcano plot shows the difference between HLA allele frequencies in the cohorts. The x-axis denotes the decimal logarithm of the odds ratio of the given allele carriers and non-carriers between different cohorts (HE = healthy exposed, CP = convalescents, HD = bone marrow donors, n=2210). Y-axis denotes the negative decimal logarithm of the p-value. P-value = 0.05 is depicted by the dotted line (Fisher exact test; statistically significant values are annotated).
